# The tandem Agenet domain of fragile X mental retardation protein interacts with FUS

**DOI:** 10.1038/s41598-017-01175-8

**Published:** 2017-04-19

**Authors:** Qingzhong He, Wei Ge

**Affiliations:** 1 National Key Laboratory of Medical Molecular Biology & Department of Biochemistry and Molecular Biology, Institute of Basic Medical Sciences, Chinese Academy of Medical Sciences, Beijing, China; 2 National Key Laboratory of Medical Molecular Biology & Department of Immunology, Institute of Basic Medical Sciences, Chinese Academy of Medical Sciences, Beijing, China

## Abstract

The tandem Agenet domain (TAD) of fragile X mental retardation protein (FMRP) protein is considered to be a member of the methyl-lysine-binding Tudor domain “Royal family”. Several groups have reported that the TAD binds with methylated histones and plays a role in DNA damage responses. FMRP is a RNA-binding protein predominantly resident in cytoplasm. Therefore, in this study, we identified DDX5, FUS, EWSR1 and LSM14A as TAD-interacting proteins sensitive to F32L and/or Y96L mutation by pull-down assays and mass spectrometry. We also showed that the interaction is potentially mediated by RGG/RG motifs. Furthermore, when FMRP was knocked-down, translocation of exogenously expressed wild-type FUS and disease-related mutant R514G was observed. This study may provide a novel insight into FMRP involvement in the intracellular localization of FUS and pathology of FUS-related amyotrophic lateral sclerosis.

## Introduction

Silencing or mutation of the human *FMR1* gene underlies the molecular mechanism of fragile X syndrome (FXS)^1, 2^. This inherited mental retardation affects people with an incidence of approximately 1/4,000 men and 1/8,000 women^3^. *FMR1* encodes the fragile X mental retardation protein (FMRP), which is a RNA-binding protein that shuttles between the nucleus and cytoplasm, and is a component of mRNA ribonucleoprotein (mRNP) particles^4–6^. FMRP acts as a repressive regulator of translation probably by forming stress granules and/or stalling ribosomes on mRNA^7, 8^.

In addition to the RNA-binding domains KH0, KH1, KH2 and RGG/RG, FMRP possesses a tandem Agenet domain (TAD) at the extreme N-terminus^9, 10^. This domain comprises two plant Agenet-like domains, Agenet1 and Agenet2, which have been identified bioinformatically as members of the methyl-lysine/arginine-recognizing Tudor domain “Royal Family”^10^. In 2006, Ramos *et al*. revealed that the TAD binds with methyl-lysine, but not methyl-arginine or unmethylated amino acids in NMR assays^11^. However, the ligands were not in a peptide context. Subsequently in 2010, Adams–Cioaba *et al*. showed that the TADs of the FMRP homologs FXR1 and FXR2 bind to histone H4K20 peptides, with a preference for trimethylation^12^. This indicated the potential of FMRP as a methylated histone-binding protein. In 2014, Alpatov *et al*. showed that the TAD of FMRP binds to mono-nucleosomes *in vitro* in an interaction that is mediated by the methylated lysines in the histone H3^13^. They also revealed that FMRP plays a role in the replication stress response^13^; this was confirmed in *Drosophila* by another group^14^. These studies suggested a chromatin-dependent aspect to the function of FMRP, although convincing evidence of a direct contact between FMRP and chromatin remains to be obtained^15^.

In the steady-state, FMRP is a predominantly cytoplasmic protein^16^. The rarity of histones in cytoplasm suggests that the majority of FMRP molecules do not interact with histone proteins. Here, we report that the TADs of FMRP bind to the protein FUS via an interaction that is potentially mediated by the RGG/RG motifs. Knockdown of FMRP interferes with the formation of cytoplasmic aggregates by FUS disease-related mutant R514G. Our research may provide a new insight into the function of FMRP and the pathology of FUS-related amyotrophic lateral sclerosis (ALS).

## Results

**Figure 1:**
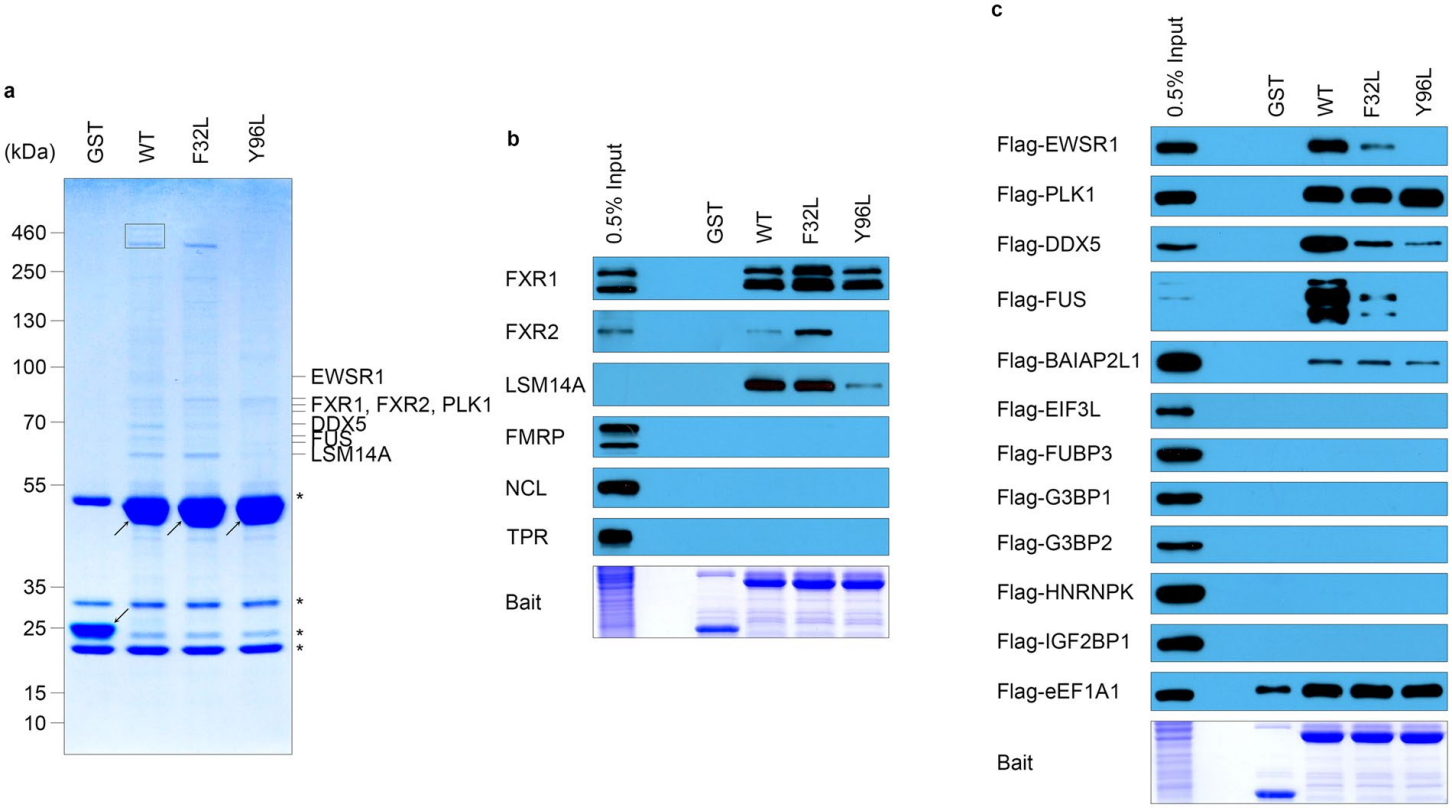
TAD of FMRP interacts with various proteins involved in RNA metabolism. (**a**) Interactors with FMRP-1-200 WT, F32L and Y96L were resolved in gels stained by Coomassie Brilliant Blue and identified by mass spectrometry. Proteins identified in the rectangle are listed in Supplementary Table 1. Asterisks indicate non-specific bands binding to glutathione agarose. Arrows indicate the bait used in the GST pull-down assay. Levels of the entire image were adjusted to reveal faint bands. The original image is shown in Supplementary Figure 1a (**b**,**c**), Interactors with FMRP-1-200 were validated in pull-down assays with endogenous (**b**) or exogenous (**c**) proteins by immunoblotting.

The interactors with the TAD of FMRP were investigated in GST pull-down assays with HEK293T cell lysates and recombinant GST-FMRP-1-200 proteins. Mass spectrometry (MS) and immunoblotting analyses revealed that FMRP-1-200 interacted with FXR1, FXR2, LSM14A, EWSR1, PLK1, DDX5, FUS and BAIAP2L1 (Fig. 1). Its putative methyl-binding pocket mutants F32L and Y96L bound FXR1, Flag-PLK1 and Flag-BAIAP2L1 with similar affinity compared with the wild-type, which suggests the mutations do not inactivate all functions of FMRP-1-200. Among the interactors, LSM14A, EWSR1, DDX5 and FUS were sensitive to the mutations, indicating that their interaction with TAD is potentially mediated by protein methylation. FMRP-1-200 possesses a potentially RNA-binding KH0 domain in addition to the TAD; therefore, RNase A was added to the pull-down assays to exclude any interaction possibly mediated by RNA. No attenuation of the pre-existing interactor bands was observed following RNase A treatment. Instead, at least three novel signals emerged, which were probably caused by exposing RNA-masked binding sites (Supplementary Figure 1).

As the mild lysis buffer barely disrupted the nuclei, few histones were engaged in the pull-down systems described (data not shown). To assess the interaction of TAD with the histones, *in vitro* pull-down assays and chromatin immunoprecipitation-cloning (ChIP-cloning) were performed.

GST pull-down assays showed that FMRP-1-200 bound to acid-extracted HeLa bulk histones rather than *E*. *coli*-expressed recombinant histone octamer. Furthermore, mutations putatively disrupting methyl-binding (F32L and Y96L) or salt bridges between the two Agenet domains [E7K and E66K, refer to ref. 12] abolished the binding (Supplementary Figure 2a–c). These results indicated that the interaction is dependent on post-translational modifications (PTMs) of histones, and the two Agenet domains within the TAD function in a synergic manner. However, FMRP-1-200 pulled down comparable amounts of both H2B/H2A and H3/H4 in this pull-down system of physiological ionic strength, suggesting a weak selectivity between core histones (Supplementary Figure 2d).

Based on these observations, we hypothesized that histone PTMs influence the interaction with FMRP-1-200 in a site-specific manner, and attempted to identify these sites with a peptide array (Supplementary Figure 3 and Supplementary Table 2) and peptide pull-down assays (Supplementary Figure 4a–f). However, no modification site showing a high affinity interaction with the FMRP N-terminus was discovered in either of the assays, despite testing several buffer formulations and protein variants.

Eventually, we employed ChIP-cloning assays to evaluate the capacity of endogenous FMRP to bind to chromatin *in vivo*. Two different FMRP antibodies were used in the ChIP-cloning assay, but few DNA was co-precipitated with endogenous FMRP (Supplementary Figure 5).

Based on these results, we questioned the function of FMRP is a histone-binding protein, especially that view that the binding is mediated by specific methylated lysine residues. The N-terminus of FMRP is acidic (FMRP-1-120 pI, 4.83 and FMRP-1-200 pI, 5.90, calculated using the ExPASy Compute pI/Mw tool). The binding of FMRP to bulk histones rather than histone peptides can be explained on the basis of electrostatic attraction. However, the pull-down assays with recombinant histone octamer and FMRP mutants indicated protein methylation is critical to the interaction. One possibility is that the two Agenet domains bind two separate histone methylation sites simultaneously resulting in a synergistic increase on the affinity.

**Figure 2:**
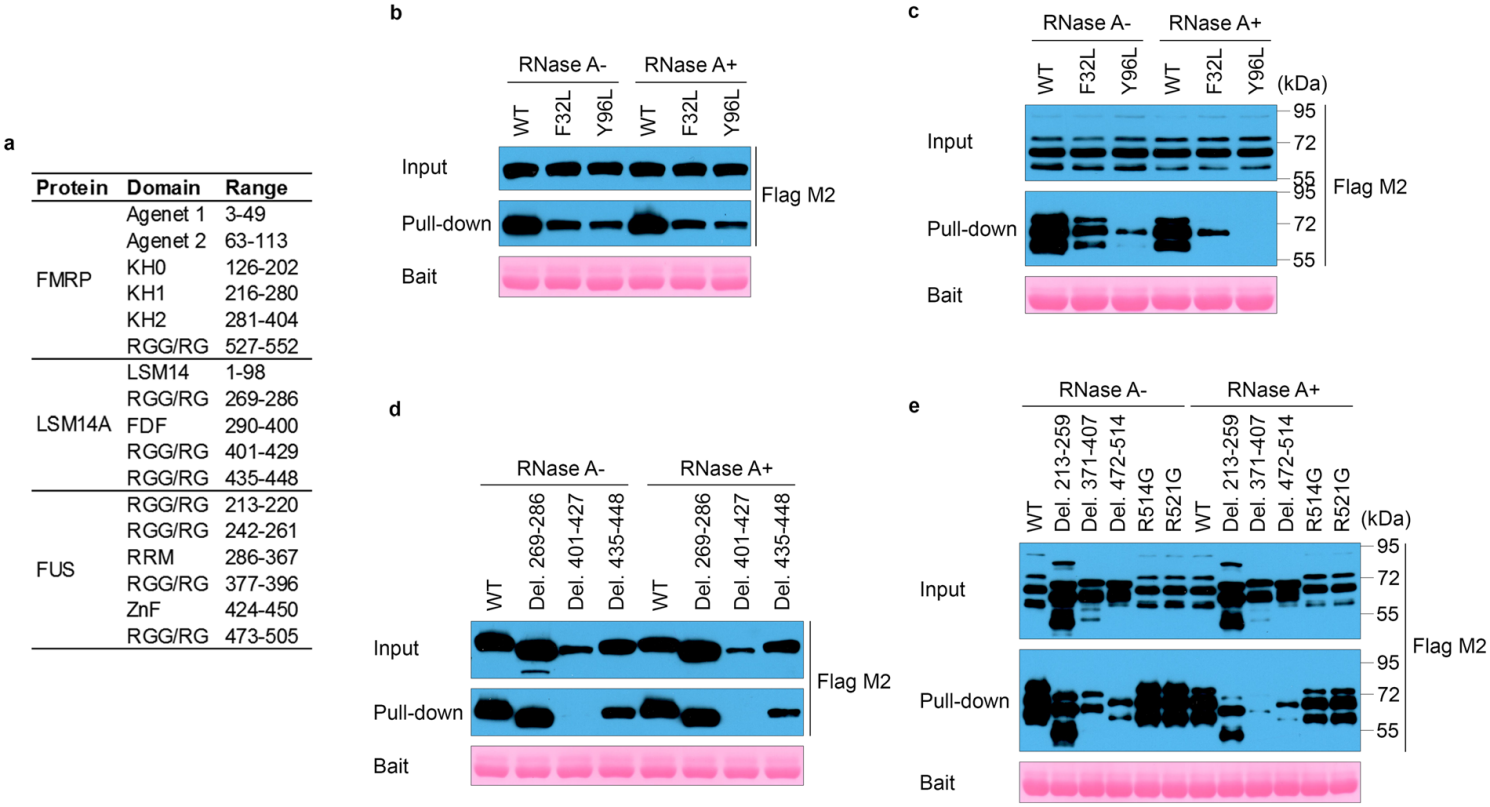
TAD of FMRP interacts with LSM14A and FUS. (**a**) Functional domains/motifs in FMRP, LSM14A and FUS proteins. (**b**,**c**) Mutations in potential methyl-binding pockets of the TAD of FMRP (GST-FMRP-1-200-F32L/Y96L) attenuate the interaction with exogenously expressed Flga-LSM14A (**b**) or Flag-FUS (**c**) in GST pull-down assays with HEK293T lysate. The involvement of RNA is excluded by RNase A treatment. (**d**,**e**) Deletion of RGG/RG motifs in LSM14A (**d**) or FUS (**e**) differentially attenuates the interaction with the TAD of FMRP. ALS-related mutations of FUS (R514G and R521G) interact with the TAD of FMRP to a similar extent compared with the wild-type. The calculated molecular weight of human FUS is 53.4 kDa. When over-expressed in cells, Flag-FUS presents mainly as two or three bands between 55 kDa and 72 kDa (**c**,**e**) which is possibly due to diverse posttranslational modifications to this protein.

After failing to elucidate the details of the FMRP-histone interaction, we re-examined the interactors identified in MS (Fig. 1). Intriguingly, the four proteins sensitive to FMRP F32L and/or Y96L mutations, DDX5, EWSR1, FUS and LSM14A, all contain methylated RGG/RG motifs (Fig. 2a), which can be recognized by some Tudor domains^17^. The interaction with DDX5 has been reported in *Drosophila* ^18^. FUS and EWSR1 are both members of the FET family^19^, and the interaction between FMRP and FUS was recently reported by Blokhuis *et al*.^20^. We then focused on the interaction of FMRP TAD with FUS and LSM14A.

We first mapped the segments/residues involved in the FMRP-FUS/LSM14A interaction. As shown in Fig. 2, FMRP mutations F32L and Y96L weakened the interaction with FUS and LSM14A (Fig. 2b,c). Furthermore, single deletion of RGG/RG motifs in FUS or LSM14A attenuated the interaction with FMRP. For LSM14A, residues 401–427 seems to be the major segment responsible for the interaction with FMRP (Fig. 2d), while for FUS, deleting any of the RGG/RG motifs reduces the amount of proteins pulled down (Fig. 2e). This raised the possibility that FMRP is involved in both monovalent and multivalent interactions with proteins. In addition, the ALS-related mutations R514G and R521G of FUS did not influence the interaction with FMRP (Fig. 2e).

FUS is a nuclear protein. When mutations such as R514G and R521G disrupt its nuclear localization signal, FUS accumulates in the cytoplasm and forms RNA-containing granules as precursors of cytoplasmic plaques discovered in some ALS patients^21^. Notably, FMRP also forms stress granules under heat or oxidative stress^7^. Therefore, we speculated that FMRP plays a role in FUS plaque formation. LSM14A was also reported to partially localize in stress granule^22^; however, in our immunofluorescence assays, there were no significant changes in the staining pattern of endogenous LSM14A following heat treatment, with main localization thought to be processing body (Supplementary Figure 6).

**Figure 3:**
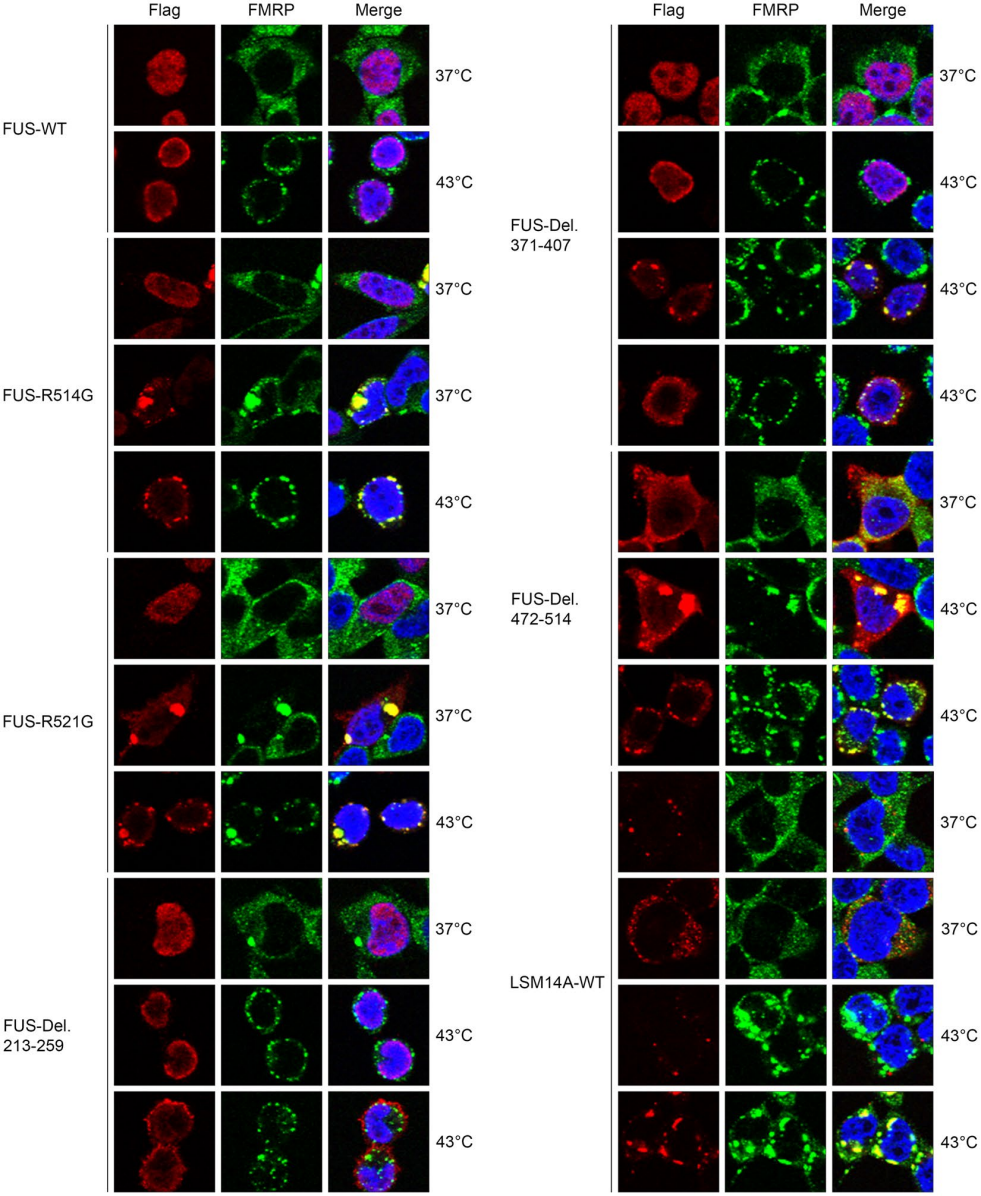
FUS and LSM14A forms stress granules with FMRP under heat stress. Wild-type Flag-FUS and its mutants, and Flag-LSM14A were transiently expressed in HEK293T cells, which were then incubated at 37 °C or 43 °C. R514G and R521G mutants of FUS formed cytoplasmic stress granules co-localizing with endogenous FMRP under heat stress, and some of the cells transfected with the R514G or R521G mutants presented cytoplasmic aggregates even at 37 °C. FUS RGG/RG motif deletion mutants Del. 213-259 and Del. 371-407 also formed stress granules under heat stress; however, co-localization with FMRP was absent in some cells. FUS Del. 472-514 localized predominantly in the cytoplasm and consistently formed granules with FMRP. Overexpressed wild-type LSM14A localized mainly in P bodies with occasional scattering in the cytoplasm. Under heat stress, LSM14A localized in stress granules in some cells.

When FUS and its mutants were expressed exogenously in HEK293T cells with N-terminal Flag tags, wild-type FUS and its mutants R514G, R521G, Del. 213-259, Del. 371-407 were localized mainly in the nucleus, with occasional aggregates formed with endogenous FMRP. Following heat stress, cytoplasmic translocation was observed with the mutants, which formed stress granules that co-stained with FMRP; however, co-localization with FMRP was not observed in some cells transfected with Del. 213-259 and Del. 371.407 (Fig. 3 and Supplementary Figure 7), which is consistent with the pull-down results in Fig. 2e. FUS mutant Del. 472-514 is predominantly localized in the cytoplasm, even in the protrusions. Although pull-down assay showed that this mutant interacts weakly with FMRP, the co-localization with FMRP in aggregates or stress granules was almost perfect (Fig. 3 and Supplementary Figure 7). Localization of endogenous LSM14A in stress granules is rare, while co-localization of overexpressed Flag-LSM14A with FMRP in the granules is quite frequent (Fig. 3).

**Figure 4:**
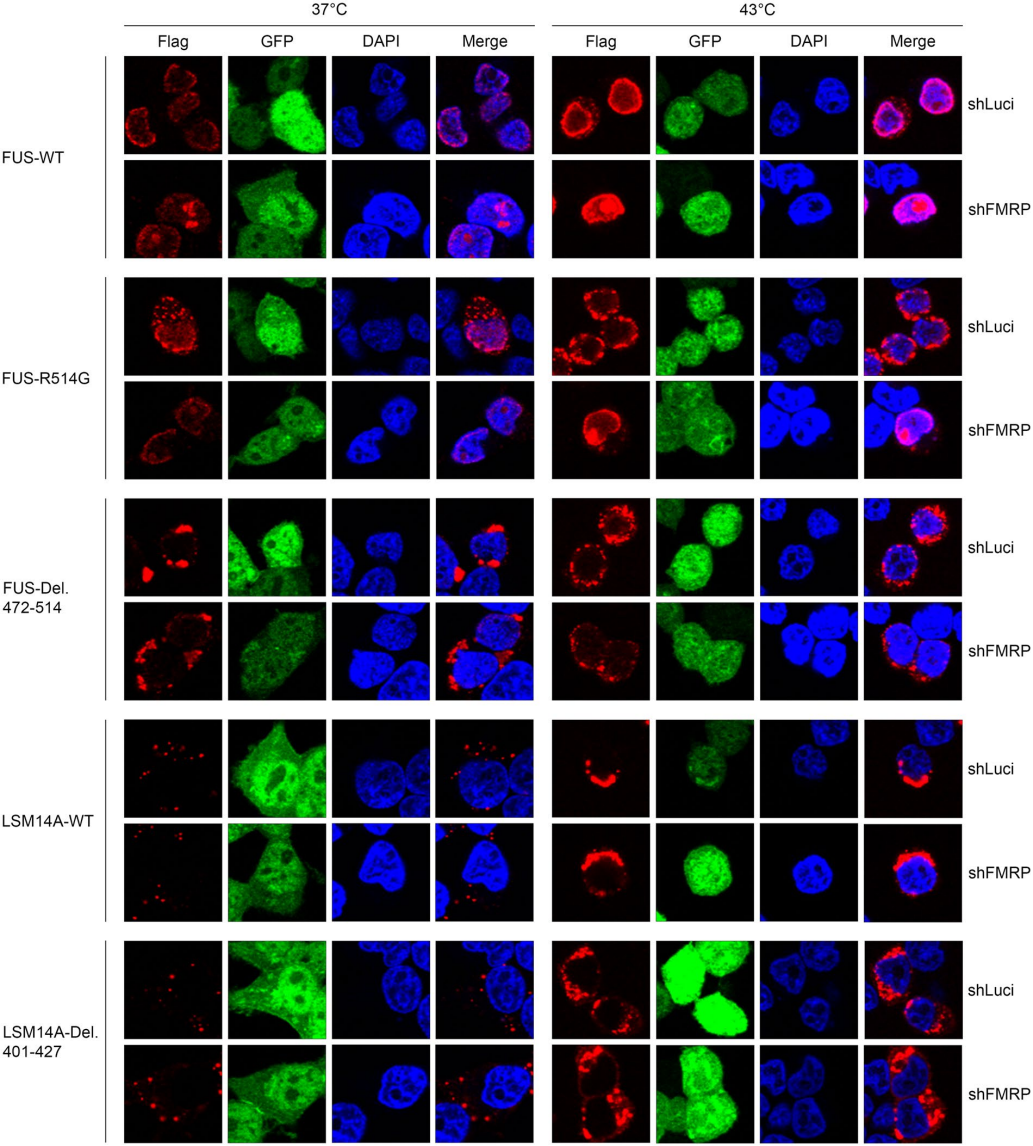
Knockdown of FMRP led to nuclear sequestration of FUS. GFP-expressing lentivirus vector was used to knockdown endogenous FMRP in HEK293T cells (shLuci: shRNA targeting *Gaussia* luciferase used as an unrelated negative control). Flag-tagged proteins were transiently transfected and cells were then subjected to heat stress. For wild-type FUS and the ALS-related mutant R514G, protein was concentrated in nuclear regions of weak DAPI staining which are thought to be nucleoli, and cytoplasmic aggregates were greatly reduced. For wild-type LSM14A and LSM14A Del. 401-427, no obvious change was observed following FMRP knockdown.

Furthermore, we investigated the mechanism by which FMRP participates in the formation of FUS plaques by knockdown of the endogenous FMRP (for knockdown efficiency, see Supplementary Figure 8), and overexpression of FUS and LSM14A, and their mutants in in HEK293T cells. When FMRP was knocked-down, nuclear wild-type FUS and R514G accumulated in the nucleolus. And the cytoplasmic translocation of R514G and Del. 472-514 was greatly reduced under heat stress (Fig. 4 and Supplementary Figure 9). However, translocation of LSM14A and its mutant Del. 401-427 was not observed.

## Discussion

In this study, GST pull-down assays showed that the TAD interacts with bulk histones, but not with recombinant histone octamer; however, we were unable to map the precise binding sites and identified its poor preference for H3/H4 or H2A/H2B. Considering that FMRP is an exclusively cytoplasmic protein in the steady-state and the acidic pI of its TAD, we tend to regard the interaction between the TAD and bulk histones as an artifact. At least, we do not think majority of the FMRP molecules in cells are engaged in the interaction with chromatin, a view that is supported by our ChIP-cloning results.

Two Agenet domains reside in the TAD of FMRP, each of which is thought to have a methyl-binding pocket. In 2002, researchers used NMR spectroscopy to demonstrate that the TAD binds methylated lysine, but not methylated arginine or unmethylated amino acids, with NMR assays. However, this binding investigation was not performed in a peptide context. Our pull-down assays with cell lysates showed that the TAD of FMRP interacts with FUS and LSM14A in an interaction that can be attenuated by methyl-binding pocket mutations and RGG/RG deletion mutations. We conclude that FMRP recognizes methylated RGG/RG motifs, although with a much lower specificity than that of other members in “Royal family”, which may provide an adaption to the sequence variation of RGG/RG motifs.

In this study, we also showed that FMRP co-localized with FUS mutant R514G aggregates. Furthermore, FMRP knockdown resulted in greatly reduced R514G cytoplasmic translocation and aggregate formation as well as altered nuclear distribution. These results indicate that endogenous FMRP sequestration is probably involved in the pathology of ALS. It should be noted that, under physiological conditions, FUS exists predominantly as a nuclear protein, while FMRP is exclusively cytoplasmic. This raises the question of when and where the proteins interact. One possibility is that FUS captures nascent transcripts from Pol II and relays them to FMRP. Alternatively, in some cells, such as neurons, FUS may interact with FMRP in dendrite spines^23^.

## Materials and Methods

### Cell lines and Antibodies

The HEK293T cell line was a kind gift from Prof. Zhang Ye.

Anti-FXR1, anti-FXR2, anti-NCL and anti-FMR1 antibodies were purchased from MBL (Nagoya, Japan). Anti-LSM14A and anti-Flag antibodies were purchased from Sigma–Aldrich (St. Louis, MO, USA). Anti-FMRP and anti-TPR antibodies were purchased from Abcam (Cambridge, MA, USA).

### Plasmids

To express recombinant GST-FMRP-1-200, the FMRP coding sequence (CDS; NM_002024.5) corresponding to amino acid M1 to M200 and a primer-introduced stop codon was inserted into the EcoR I and Sal I sites of pGEX-4T-1. F32L and Y96L mutant plasmids were constructed by site-directed mutagenesis of pGEX-4T-1-FMRP-1-200.

Plasmids encoding N-terminal Flag-tagged EWSR1 (NM_013986.3), PLK1 (NM_005030.4), DDX5 (NM_004396.3), FUS (NM_004960.3), BAIAP2L1 (NM_018842.4), EIF3L (NM_016091.3), FUBP3 (NM_003934.1), G3BP1 (NM_005754.2), G3BP2 (NM_203505.2), HNRNPK (NM_002140.3), IGF2BP1 (NM_006546.3), LSM14A (NM_001114093.1), eEF1A1 (NM_001402.5) were purchased from PolePolar Biotechnology Co., LTD (Beijing, China). The backbones are all pcDNA3.1(+)-Flag.

To knock down FMRP in HEK293T cells, a lentivirus vector was constructed with pLV-U6 (a kind gift from Prof. Depei Liu, constructed by Dr. Lei Li and Dr. Lu Lu). The target sequence was CGAGATTTCATGAACAGTTTAT and the unrelated sequence CAACAAGATGAAGAGCACCAA targeting *Gaussia* luciferase was used as a negative control.

### Recombinant protein expression and purification

To express recombinant proteins, E. coli strain BL21 bearing the relevant plasmids was grown at 37 °C to an absorbance at 600 nm of 0.6. Protein expression was induced by culturing overnight at 16 °C after the addition of 0.4 mM isopropyl β-D-1-thiogalactopyranoside (IPTG).

Bacteria were pelleted by centrifugation of 3900 rpm and suspended in 50 mM Tris-HCl, pH 7.4, 500 mM NaCl, 10% glycerol, 1% Triton X-100, 1 mM DTT and 1 mM PMSF. Bacterial suspensions were sonicated and centrifuged to remove bacterial debris and insoluble proteins and glutathione Sepharose 4B was added to the supernatant. After a 2-hour incubation with gentle rotation at 4 °C, beads were pelleted and washed sequentially with the following buffers: (1) 50 mM Tris-HCl, pH 7.4, 500 mM NaCl, 10% glycerol, 0.5% Triton X-100; (2) 50 mM Tris-HCl, pH 7.4, 500 mM NaCl, 10% glycerol; and (3) 50 mM Tris-HCl, pH7.4, 100 mM NaCl, 10% glycerol. Protein was eluted with 10 mM reduced glutathione, aliquoted and stored at 80 °C.

### GST pull-down

For each GST pull-down sample, 90% confluent cells in a 10-cm dish were lysed in 1-ml lysis buffer (100 mM NaCl, 20 mM Tris-HCl, pH 7.4, 5 mM MgCl_2_, 0.5% Igepal CA-630, and 1× protease inhibitor cocktail without EDTA). For isolation of exogenous protein, cells were harvested at 36 h post-transfection. For RNA-free samples, RNase A was added to a final concentration of 0.5 mg/ml and the enzyme buffer was used as control. After a 1-h incubation with gentle rotation at 4 °C, lysates were centrifuged at 20,000× g and supernatant was added to 20-μl glutathione Sepharose 4B pre-coupled with 20-μg GST-tagged protein. After a 4-h incubation with gentle rotation at 4 °C, the beads were pelleted by centrifugation and washed three times with 1-ml lysis buffer. After washing, the beads were mixed with 50-μl 1.6× Laemmli SDS-PAGE loading buffer and boiled for electrophoresis.

### In-gel digestion and protein identification

Briefly, following SDS-PAGE, gels were stained with Coomassie Brilliant Blue, and the protein bands were cut off into 1 mm^3^ cubes. After dehydration, the gel cubes were treated with DTT and IAA, and then digested with trypsin. Peptides were extracted from gels with ammonium bicarbonate/acetonitrile, dried and re-dissolved in 0.1% formic acid for MS analysis.

The peptides were analyzed using a Thermo Fisher Orbitrap Fusion™ Tribrid™ Mass Spectrometer. The data were collected by Xcalibur 2.1.2 and further interpreted with Proteome Discoverer 1.4.

### Immunofluorescence

Cells were seeded on gelatin-coated glass slides in 6-well plates. Before harvesting, plates were floated for 30 min in a water bath maintained at 37 °C or 43 °C. Cells were fixed with fresh 4% paraformaldehyde, permeabilized with 0.1% Triton X-100 in PBS and blocked with 5% goat serum in PBS. Blocked cells were incubated with relevant primary antibodies for 2 h at room temperature and then, with relevant secondary antibodies for 1 h. Slides were counterstained with DAPI in the mounting medium.

## Electronic supplementary material

Supplementary Figures and Tables
