# Supplementary material for: The tandem Agenet domain of fragile X mental retardation protein interacts with FUS
Source: Sci Rep. 2017 Apr 19;7:962. doi: 10.1038/s41598-017-01175-8 (PMC5430443; doi:10.1038/s41598-017-01175-8)
Supplement: Supplementary file 1 — Supplementary Figures and Tables [file 41598_2017_1175_MOESM1_ESM.pdf]

Title

The tandem Agenet domain of fragile X mental retardation protein interacts with FUS

Author names and affiliations

Qingzhong He[1], Wei Ge[2*]

1 National Key Laboratory of Medical Molecular Biology & Department of Biochemistry and Molecular Biology, Institute of Basic Medical Sciences, Chinese Academy of Medical Sciences, Beijing, China

2 National Key Laboratory of Medical Molecular Biology & Department of Immunology, Institute of Basic Medical Sciences, Chinese Academy of Medical Sciences, Beijing, China

Supplementary Figures

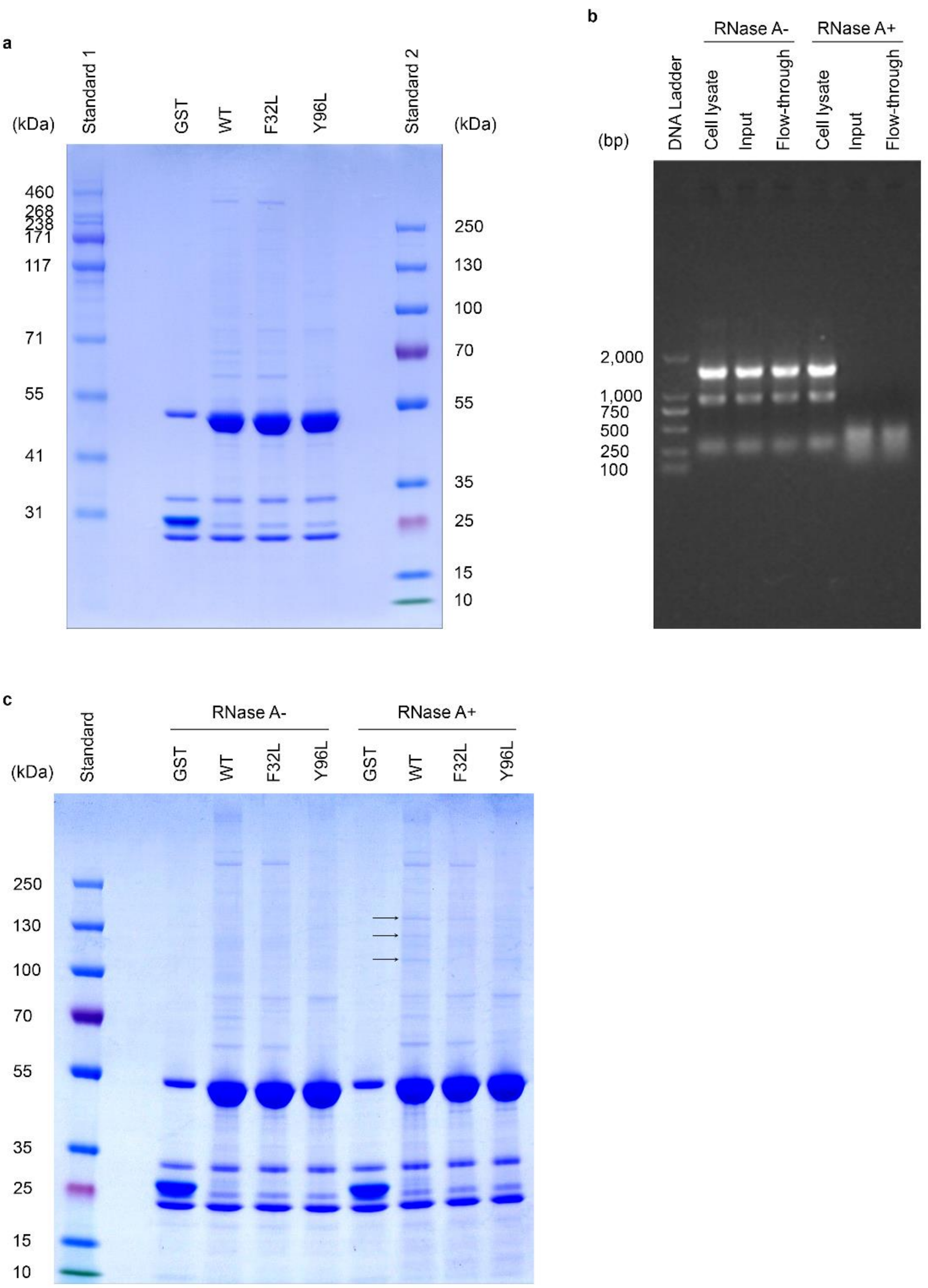

Supplementary Figure 1 FMRP-1-200 interacts with several proteins in an RNA-independent manner. a, Original image of Figure 1a. b, RNase A treatment degraded almost all RNAs in the GST pull-down input. “Cell lysate”, fresh cell lysate without any treatment. “Input”, cell lysate treated by RNase A or the enzyme buffer, used

in the GST pull-down. “Flow-through”, GST pull-down flow-through of the “input”. c, Interaction with FMRP-1-200 was not attenuated by treatment with RNase A. Instead, several new bands (indicated by arrows) emerged with the wild-type bait after the treatment.

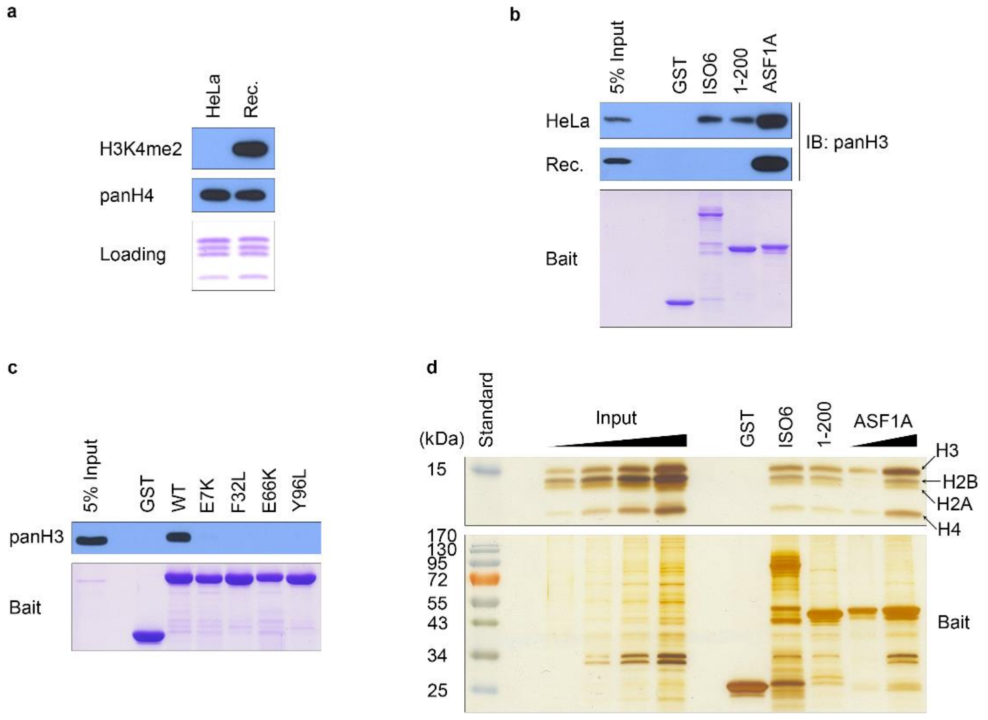

Supplementary Figure 2 FMRP-1-200 interacts with histones in vitro. a, Histones used in GST pull-down assays. HeLa, acid-extracted bulk histones from HeLa cells, with post-translational modifications (PTMs). Rec., recombinant histone octamer with no PTMs. b, FMRP-1-200 interacts with HeLa histones but not recombinant histones, which suggests the interaction is PTM-dependent. c, Single mutations on putative salt bridges (E7K and E66K) and methyl-binding pockets (F32L and Y96L) abolish the interaction with histones. d, Silver staining revealed that FMRP-1-200 showed no apparent preference for the H3-H4 tetramer or H2B-H2A dimer, compared with chaperone ASF1A. ISO6: Full-length GST-tagged human FMRP isoform 6 (GenBank: NP_001172004.1).

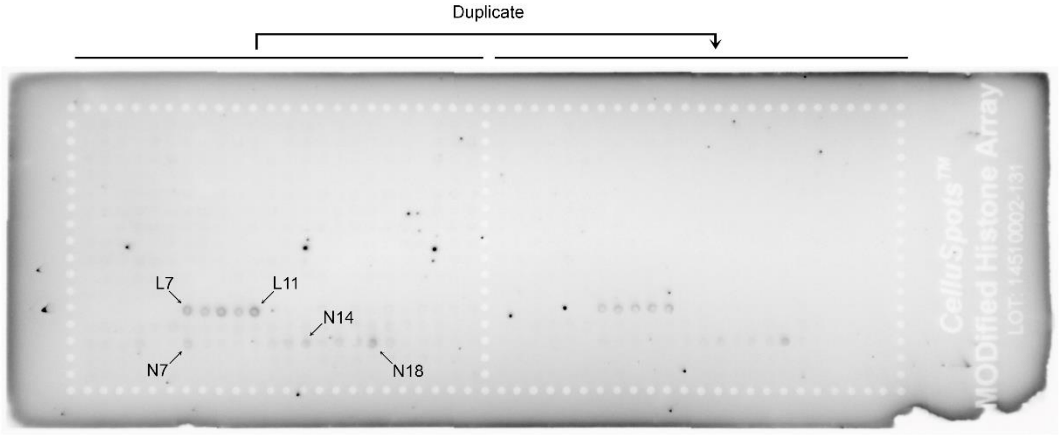

Supplementary Figure 3 GST-FMRP-1-200 showed a weak interaction with Dot L7-L11, N7, N14 and N18 on the MODified™ Histone Peptide Array of Active Motif®. Please refer to Supplementary Table 2 for the corresponding peptide sequences of the dots. The peptide array was a kind gift from Prof. Guohong Li. We followed the manufacturer's protocol to perform the assay. Briefly, 15-ml 1-μM purified GST-FMRP-1-200 was incubated with the array blocked by skim milk. After the incubation, the array was washed and detected with anti-GST and a HRP-conjugated secondary antibody by chemiluminescent substrate.

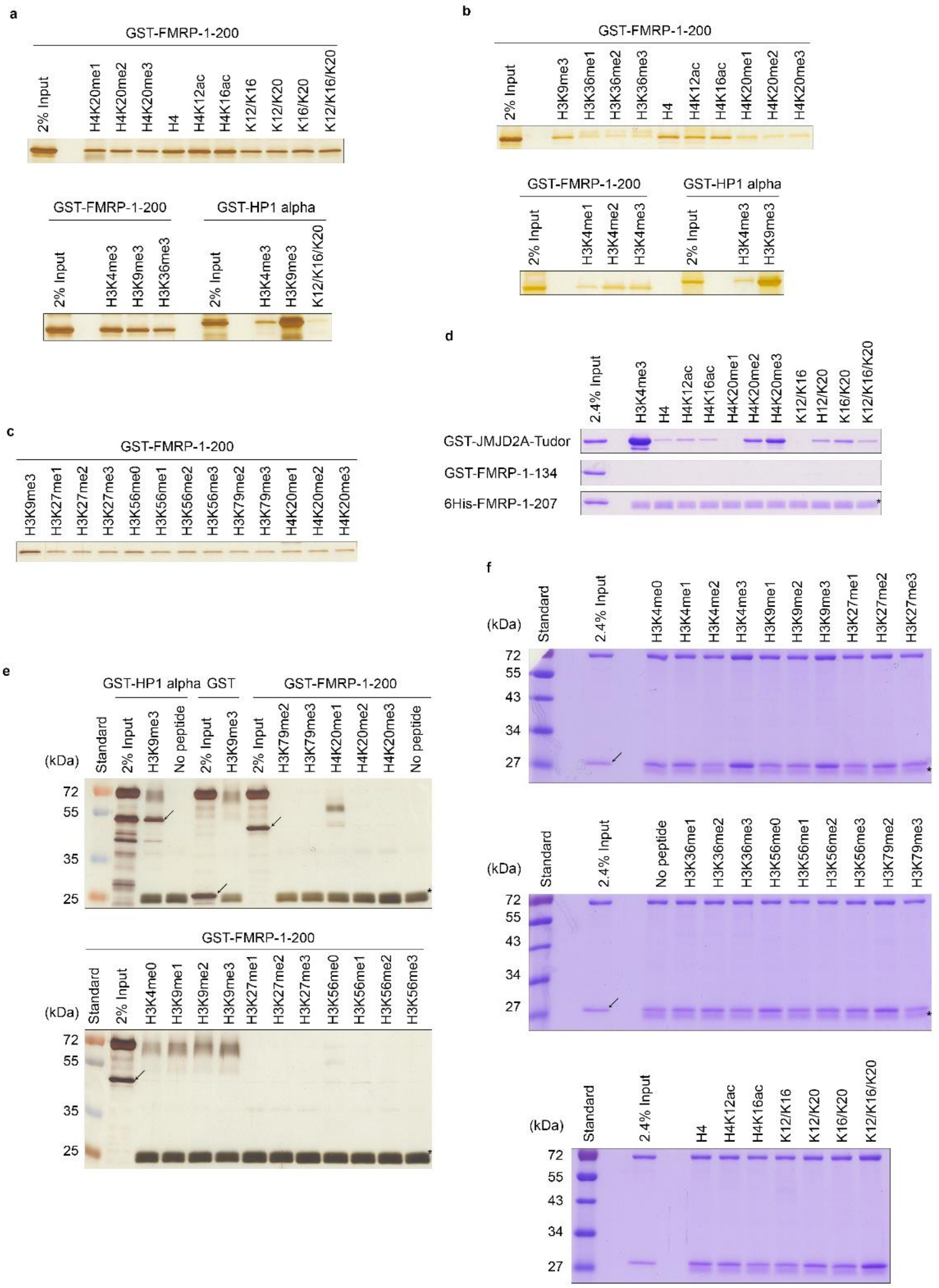

Supplementary Figure 4 The TAD of FMRP showed no strong interaction with lysine-methylated histone peptides in peptide pull-down assays. a, GST-FMRP-1-200 showed weak/negligible interaction with modified histone peptides in pull-down assays. GST-HP1 alpha represents the positive control. Pull-down system: 2 μg peptide vs. 10

µg protein in 10 mM Tris-HCl, pH 7.4, 150 mM NaCl, 0.05% Tween® 20, 0.04 mg/ml BSA. b, GST-FMRP-1-200 showed weak/negligible interactions with modified histone peptides in pull-down assays. GST-HP1 alpha represents the positive control. Pull-down system: 2 µg peptide vs. 10 µg protein in 10 mM Tris-HCl, pH 7.4, 150 mM NaCl, 0.05% Tween® 20, 0.04 mg/ml BSA. c, GST-FMRP-1-200 showed no preference among H3K27me1/2/3, H3K56me0/1/2/3, H3K79me2/3 and H4K20me1/2/3. Pull-down system: 2 µg peptide vs. 10 µg protein in 50 mM Tris-HCl, pH 7.4, 100 mM NaCl, 0.5% Triton™ X-100, 0.03 mg/ml BSA. d, GST-FMRP-1-134 and 6His-FMRP-1-207 showed no binding with modified H4 peptides. GST-JMJD2A-Tudor vs. H3K4me3 was used as the positive control. Pull-down system: 20 mM Tris-HCl, pH 8.0, 200 mM NaCl, 0.01% IGEPAL® CA-630, 10% glycerol. e, GST-FMRP-1-200 showed weak/negligible interactions with modified histone peptides in pull-down assays. GST-HP1 alpha was used as the positive control. GST was used as the negative control. Pull-down system: 50 mM Tris-HCl, pH 7.4, 300 mM NaCl, 0.05% IGEPAL® CA-630, 0.03 mg/ml BSA. f, 6His-FMRP-1-207 showed weak/negligible interactions with modified histone peptides in pull-down assays. Pull-down system: 20 mM Tris-HCl, pH 7.4, 150 mM NaCl, 0.04 mg/ml BSA. Arrows indicate the prey proteins. Asterisks indicate bands derived from streptavidin beads. K12/K16, H4G11-T30 (K12ac, K16ac). K12/K20, H4G11-T30 (K12ac, K20me3). K16/K20, H4G11-T30 (K16ac, K20me3). K12/K16/K20, H4G11-T30 (K12ac, K16ac, K20me3).

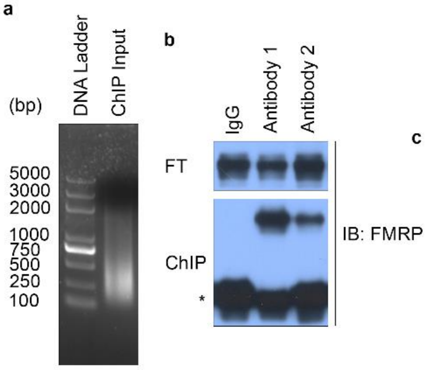

c

Dish	Colony No.
IgG	15
Antibody 1	5
Antibody 2	2
H3	>150

Supplementary Figure 5 ChIP-cloning assay showed that few DNAs co-precipitated with endogenous FMRP in Neuro-2a cells. a, Chromatin fragments of approx. 250 bp were used as the ChIP input. b, Antibody 1 (anti-FMRP) and Antibody 2 (anti-FMR1) precipitated endogenous FMRP in ChIP assays. Asterisk indicates IgG heavy chains. c, Bacterial colony numbers derived from different antibodies reflect the co-precipitation of relatively few DNAs with endogenous FMRP.

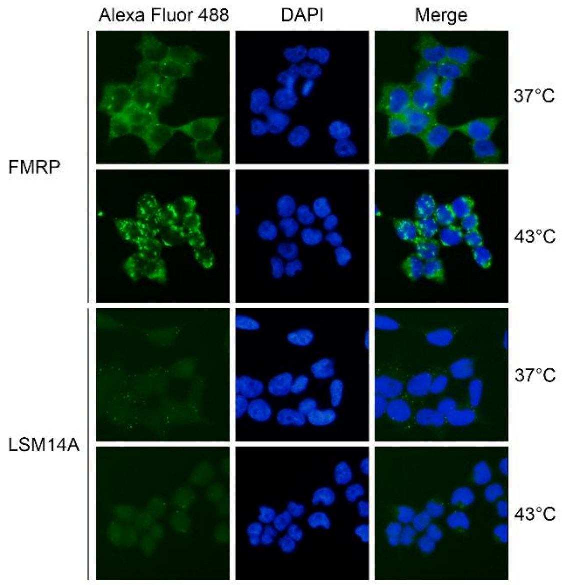

Supplementary Figure 6 Under heat stress, endogenous FMRP forms stress granules in the cytoplasm, while endogenous LSM14A resides in P-bodies. Endogenous FMRP or LSM14A was marked by indirect immunofluorescence with Alexa Fluor 488-conjugated secondary antibodies. Heat stress was applied by incubating cells at 43°C for 30 min. Images were captured under a conventional microscope.

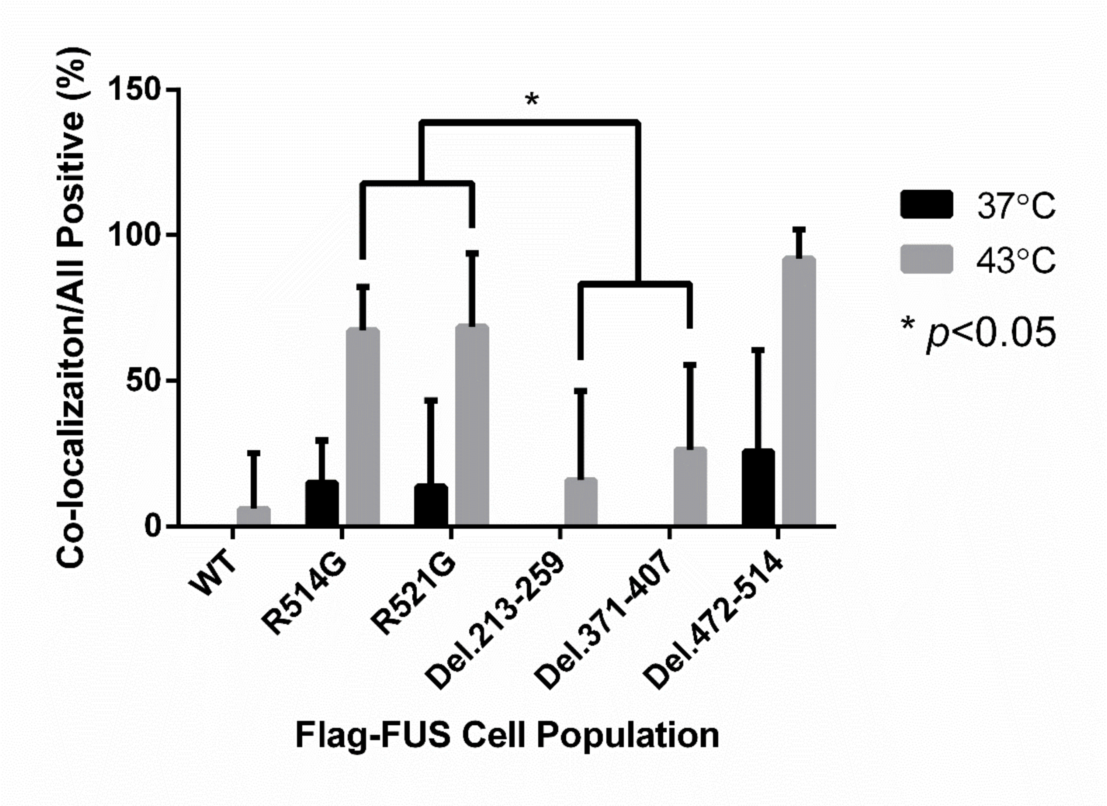

Supplementary Figure 7 When 213-259 or 371-407 was deleted, the co-localization of Flag-FUS and endogenous FMRP was attenuated under heat stress. At least 10 microscopy fields were randomly collected from each cell population transfected with Flag-FUS or its mutants. The ratio of FMRP-Flag co-localization cell number to all Flag-positive cell number was calculated. Values are shown as the means ± SD. Student's *t*-tests were performed. *P*-values less than 0.05 were considered statistically significant.

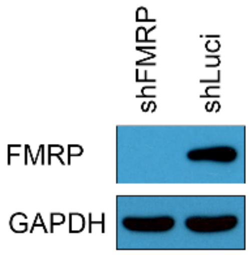

Supplementary Figure 8 Efficiency of lentivirus vector-mediated FMRP knockdown in HEK293T cells.

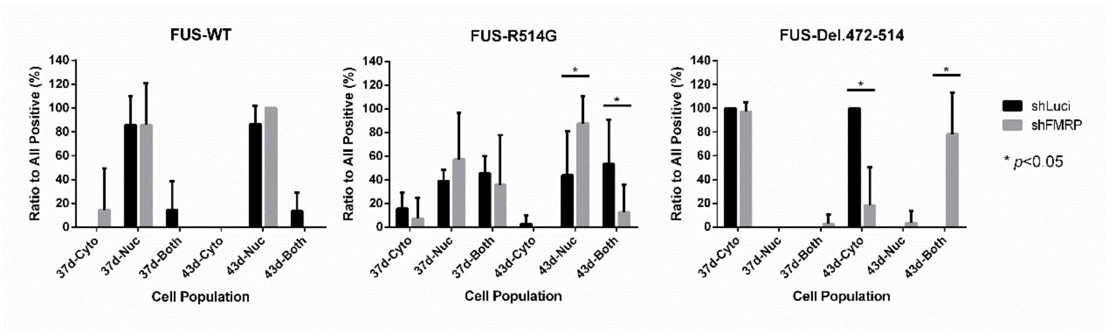

Supplementary Figure 9 FMRP knockdown enhanced the nuclear accumulation of FUS mutants under heat stress. At least six microscopy fields were collected from each cell population transfected with Flag-FUS or its mutants and treated at 37ºC (“37d”) or 43ºC (“43d”). The ratio of specifically localized cell numbers to all Flag-positive cell numbers was calculated (Cyto, cytoplasmic. Nuc, nuclear. Both, both in cytoplasm and nucleus). Values are the means ± SD. Student’s *t*-tests were performed. *P*-values <0.05 were considered statistically significant.

Supplementary Table 1 The list of proteins identified with mass spectrum in the rectangle area of SDS-PAGE in Figure 1a.

Accession	Description	Score	Coverage	Unique Peptides	MW [kDa]	calc. pI
Q5JRA6	Melanoma inhibitory activity protein 3 OS=Homo sapiens GN=MIA3 PE=1 SV=1 - [MIA3_HUMAN]	4183.31	60.88	109	213.6	4.84
P12270	Nucleoprotein TPR OS=Homo sapiens GN=TPR PE=1 SV=3 - [TPR_HUMAN]	1873.95	53.70	126	267.1	5.02
Q09666	Neuroblast differentiation-associated protein AHNAK OS=Homo sapiens GN=AHNAK PE=1 SV=2 - [AHNK_HUMAN]	867.48	51.32	153	628.7	6.15
P48634	Protein PRRC2A OS=Homo sapiens GN=PRRC2A PE=1 SV=3 - [PRC2A_HUMAN]	324.12	34.72	51	228.7	9.45
P35527	Keratin, type I cytoskeletal 9 OS=Homo sapiens GN=KRT9 PE=1 SV=3 - [K1C9_HUMAN]	290.57	60.03	24	62.0	5.24
P04264	Keratin, type II cytoskeletal 1 OS=Homo sapiens GN=KRT1 PE=1 SV=6 - [K2C1_HUMAN]	256.55	50.16	25	66.0	8.12
P15924	Desmoplakin OS=Homo sapiens GN=DSP PE=1 SV=3 - [DESP_HUMAN]	240.21	27.38	72	331.6	6.81
P49327	Fatty acid synthase OS=Homo sapiens GN=FASN PE=1 SV=3 - [FAS_HUMAN]	228.22	23.62	45	273.3	6.44
Q06787	Fragile X mental retardation protein 1 OS=Homo sapiens GN=FMR1 PE=1 SV=1 - [FMR1_HUMAN]	213.56	13.77	8	71.1	7.42
Q8IWZ3	Ankyrin repeat and KH domain-containing protein 1 OS=Homo sapiens GN=ANKHD1 PE=1 SV=1 - [ANKH1_HUMAN]	139.21	16.13	23	269.3	5.73
P68104	Elongation factor 1-alpha 1 OS=Homo sapiens GN=EEF1A1 PE=1 SV=1 - [EF1A1_HUMAN]	119.54	30.09	13	50.1	9.01
P07477	Trypsin-1 OS=Homo sapiens GN=PRSS1 PE=1 SV=1 - [TRY1_HUMAN]	102.23	7.29	2	26.5	6.51
P35908	Keratin, type II cytoskeletal 2 epidermal OS=Homo sapiens GN=KRT2 PE=1 SV=2 - [K22E_HUMAN]	100.18	43.97	18	65.4	8.00
Q9Y520	Protein PRRC2C OS=Homo sapiens GN=PRRC2C PE=1 SV=4 - [PRC2C_HUMAN]	95.60	10.32	20	316.7	9.13
P13645	Keratin, type I cytoskeletal 10 OS=Homo sapiens GN=KRT10 PE=1 SV=6 - [K1C10_HUMAN]	94.32	34.59	17	58.8	5.21
O15027	Protein transport protein Sec16A OS=Homo sapiens GN=SEC16A PE=1 SV=3 - [SC16A_HUMAN]	90.21	12.67	19	233.4	5.63
Q8IVF2	Protein AHNAK2 OS=Homo sapiens GN=AHNAK2 PE=1 SV=2 - [AHNK2_HUMAN]	77.88	14.60	22	616.2	5.36
O75179	Ankyrin repeat domain-containing protein 17 OS=Homo sapiens GN=ANKRD17 PE=1 SV=3 - [ANR17_HUMAN]	69.54	9.53	8	274.1	6.52
Q14643	Inositol 1,4,5-trisphosphate receptor type 1 OS=Homo sapiens GN=ITPR1 PE=1 SV=3 - [ITPR1_HUMAN]	67.80	8.96	14	313.7	6.04
Q14573	Inositol 1,4,5-trisphosphate receptor type 3 OS=Homo sapiens GN=ITPR3 PE=1 SV=2 - [ITPR3_HUMAN]	57.10	8.54	15	303.9	6.48
Q14571	Inositol 1,4,5-trisphosphate receptor type 2 OS=Homo sapiens GN=ITPR2 PE=1 SV=2 - [ITPR2_HUMAN]	55.94	8.22	14	307.9	6.43
P21333	Filamin-A OS=Homo sapiens GN=FLNA PE=1 SV=4 - [FLNA_HUMAN]	50.20	9.48	18	280.6	6.06
Q5T5U3	Rho GTPase-activating protein 21 OS=Homo sapiens GN=ARHGAP21 PE=1 SV=1 - [RHG21_HUMAN]	49.59	11.39	16	217.2	7.80
Q6P2Q9	Pre-mRNA-processing-splicing factor 8 OS=Homo sapiens GN=PRPF8 PE=1 SV=2 - [PRP8_HUMAN]	49.40	12.03	22	273.4	8.84
Q9BTC0	Death-inducer obliterator 1 OS=Homo sapiens GN=DIDO1 PE=1 SV=5 - [DIDO1_HUMAN]	49.03	11.65	17	243.7	7.88
P27708	CAD protein OS=Homo sapiens GN=CAD PE=1 SV=3 - [PYR1_HUMAN]	45.22	12.31	22	242.8	6.46

Q9H4A3	Serine/threonine-protein kinase WNK1 OS=Homo sapiens GN=WNK1 PE=1 SV=2 - [WNK1_HUMAN]	45.05	8.86	15	250.6	6.34
P27816	Microtubule-associated protein 4 OS=Homo sapiens GN=MAP4 PE=1 SV=3 - [MAP4_HUMAN]	44.38	15.63	13	120.9	5.43
Q92616	Translational activator GCN1 OS=Homo sapiens GN=GCN1L1 PE=1 SV=6 - [GCN1L_HUMAN]	42.14	8.09	19	292.6	7.47
P78527	DNA-dependent protein kinase catalytic subunit OS=Homo sapiens GN=PRKDC PE=1 SV=3 - [PRKDC_HUMAN]	41.99	4.82	17	468.8	7.12
O75369	Filamin-B OS=Homo sapiens GN=FLNB PE=1 SV=2 - [FLNB_HUMAN]	35.08	6.96	15	278.0	5.73
P13647	Keratin, type II cytoskeletal 5 OS=Homo sapiens GN=KRT5 PE=1 SV=3 - [K2C5_HUMAN]	34.01	19.15	6	62.3	7.74
P02533	Keratin, type I cytoskeletal 14 OS=Homo sapiens GN=KRT14 PE=1 SV=4 - [K1C14_HUMAN]	29.77	24.79	6	51.5	5.16
Q9UGU0	Transcription factor 20 OS=Homo sapiens GN=TCF20 PE=1 SV=3 - [TCF20_HUMAN]	28.12	8.93	13	211.6	9.04
Q5JSZ5	Protein PRRC2B OS=Homo sapiens GN=PRRC2B PE=1 SV=2 - [PRC2B_HUMAN]	26.23	4.85	6	242.8	8.34
Q04637	Eukaryotic translation initiation factor 4 gamma 1 OS=Homo sapiens GN=EIF4G1 PE=1 SV=4 - [IF4G1_HUMAN]	26.17	5.75	7	175.4	5.33
P02538	Keratin, type II cytoskeletal 6A OS=Homo sapiens GN=KRT6A PE=1 SV=3 - [K2C6A_HUMAN]	25.53	16.49	4	60.0	8.00
Q93008	Probable ubiquitin carboxyl-terminal hydrolase FAF-X OS=Homo sapiens GN=USP9X PE=1 SV=3 - [USP9X_HUMAN]	20.12	4.09	8	292.1	5.80
Q14444	Caprin-1 OS=Homo sapiens GN=CAPRIN1 PE=1 SV=2 - [CAPR1_HUMAN]	19.75	15.94	9	78.3	5.25
Q14839	Chromodomain-helicase-DNA-binding protein 4 OS=Homo sapiens GN=CHD4 PE=1 SV=2 - [CHD4_HUMAN]	19.19	5.81	9	217.9	5.86
Q9UBX7	Kallikrein-11 OS=Homo sapiens GN=KLK11 PE=1 SV=2 - [KLK11_HUMAN]	18.35	2.48	1	31.0	8.94
P02768	Serum albumin OS=Homo sapiens GN=ALB PE=1 SV=2 - [ALBU_HUMAN]	18.01	2.46	1	69.3	6.28
Q5VUA4	Zinc finger protein 318 OS=Homo sapiens GN=ZNF318 PE=1 SV=2 - [ZN318_HUMAN]	16.72	4.43	8	251.0	7.20
P35579	Myosin-9 OS=Homo sapiens GN=MYH9 PE=1 SV=4 - [MYH9_HUMAN]	16.66	4.08	6	226.4	5.60
Q01844	RNA-binding protein EWS OS=Homo sapiens GN=EWSR1 PE=1 SV=1 - [EWS_HUMAN]	14.93	7.16	3	68.4	9.33
Q12789	General transcription factor 3C polypeptide 1 OS=Homo sapiens GN=GTF3C1 PE=1 SV=4 - [TF3C1_HUMAN]	13.62	3.75	6	238.7	7.30
Q9C0C2	182 kDa tankyrase-1-binding protein OS=Homo sapiens GN=TNKS1BP1 PE=1 SV=4 - [TB182_HUMAN]	13.43	5.15	6	181.7	4.86
Q14966	Zinc finger protein 638 OS=Homo sapiens GN=ZNF638 PE=1 SV=2 - [ZN638_HUMAN]	13.25	3.89	6	220.5	6.38
Q9P2E9	Ribosome-binding protein 1 OS=Homo sapiens GN=RRBP1 PE=1 SV=4 - [RRBP1_HUMAN]	13.07	4.96	5	152.4	8.60
P09211	Glutathione S-transferase P OS=Homo sapiens GN=GSTP1 PE=1 SV=2 - [GSTP1_HUMAN]	11.78	9.52	1	23.3	5.64
Q8IWI9	MAX gene-associated protein OS=Homo sapiens GN=MGA PE=1 SV=3 - [MGAP_HUMAN]	10.43	1.85	4	331.6	6.79
Q9ULT8	E3 ubiquitin-protein ligase HECTD1 OS=Homo sapiens GN=HECTD1 PE=1 SV=3 - [HECD1_HUMAN]	10.30	2.61	5	289.2	5.35
Q92576	PHD finger protein 3 OS=Homo sapiens GN=PHF3 PE=1 SV=3 - [PHF3_HUMAN]	9.39	2.65	5	229.3	6.96

P35658	Nuclear pore complex protein Nup214 OS=Homo sapiens GN=NUP214 PE=1 SV=2 - [NU214_HUMAN]	7.42	2.97	5	213.5	7.47
Q6I9Y2	THO complex subunit 7 homolog OS=Homo sapiens GN=THOC7 PE=1 SV=3 - [THOC7_HUMAN]	7.20	4.41	1	23.7	5.67
P49792	E3 SUMO-protein ligase RanBP2 OS=Homo sapiens GN=RANBP2 PE=1 SV=2 - [RBP2_HUMAN]	6.97	1.80	3	358.0	6.20
P51610	Host cell factor 1 OS=Homo sapiens GN=HCFC1 PE=1 SV=2 - [HCFC1_HUMAN]	6.89	1.38	2	208.6	7.46
Q96N67	Dedicator of cytokinesis protein 7 OS=Homo sapiens GN=DOCK7 PE=1 SV=4 - [DOCK7_HUMAN]	6.83	1.21	2	242.4	6.80
Q13428	Treacle protein OS=Homo sapiens GN=TCOF1 PE=1 SV=3 - [TCOF_HUMAN]	6.72	1.55	2	152.0	9.04
P0CG48	Polyubiquitin-C OS=Homo sapiens GN=UBC PE=1 SV=3 - [UBC_HUMAN]	6.71	21.02	1	77.0	7.66
A5YKK6	CCR4-NOT transcription complex subunit 1 OS=Homo sapiens GN=CNOT1 PE=1 SV=2 - [CNOT1_HUMAN]	6.67	1.43	3	266.8	7.11
P13637	Sodium/potassium-transporting ATPase subunit alpha-3 OS=Homo sapiens GN=ATP1A3 PE=1 SV=3 - [AT1A3_HUMAN]	6.25	3.65	2	111.7	5.38
P26641	Elongation factor 1-gamma OS=Homo sapiens GN=EEF1G PE=1 SV=3 - [EF1G_HUMAN]	6.15	8.01	3	50.1	6.67
Q00839	Heterogeneous nuclear ribonucleoprotein U OS=Homo sapiens GN=HNRNPU PE=1 SV=6 - [HNRPU_HUMAN]	5.88	5.70	2	90.5	6.00
P16615	Sarcoplasmic/endoplasmic reticulum calcium ATPase 2 OS=Homo sapiens GN=ATP2A2 PE=1 SV=1 - [AT2A2_HUMAN]	5.76	2.50	2	114.7	5.34
Q86YZ3	Hornerin OS=Homo sapiens GN=HRNR PE=1 SV=2 - [HORN_HUMAN]	5.19	1.89	2	282.2	10.04
Q9P281	BAH and coiled-coil domain-containing protein 1 OS=Homo sapiens GN=BAHCC1 PE=1 SV=3 - [BAHC1_HUMAN]	4.98	1.19	2	276.8	8.81
P81605	Dermcidin OS=Homo sapiens GN=DCD PE=1 SV=2 - [DCD_HUMAN]	4.89	20.00	2	11.3	6.54
Q14204	Cytoplasmic dynein 1 heavy chain 1 OS=Homo sapiens GN=DYNC1H1 PE=1 SV=5 - [DYHC1_HUMAN]	4.70	0.54	2	532.1	6.40
Q7Z6Z7	E3 ubiquitin-protein ligase HUWE1 OS=Homo sapiens GN=HUWE1 PE=1 SV=3 - [HUWE1_HUMAN]	4.69	0.80	2	481.6	5.22
P54652	Heat shock-related 70 kDa protein 2 OS=Homo sapiens GN=HSPA2 PE=1 SV=1 - [HSP72_HUMAN]	4.62	4.54	2	70.0	5.74
O75643	U5 small nuclear ribonucleoprotein 200 kDa helicase OS=Homo sapiens GN=SNRNP200 PE=1 SV=2 - [U520_HUMAN]	4.16	0.98	2	244.4	6.06
O75376	Nuclear receptor corepressor 1 OS=Homo sapiens GN=NCOR1 PE=1 SV=2 - [NCOR1_HUMAN]	4.09	1.02	2	270.0	7.11
Q9UPU5	Ubiquitin carboxyl-terminal hydrolase 24 OS=Homo sapiens GN=USP24 PE=1 SV=3 - [UBP24_HUMAN]	3.94	0.92	2	294.2	6.14
O43252	Bifunctional 3'-phosphoadenosine 5'-phosphosulfate synthase 1 OS=Homo sapiens GN=PAPSS1 PE=1 SV=2 - [PAPS1_HUMAN]	3.71	2.24	1	70.8	6.86
Q9BY89	Uncharacterized protein KIAA1671 OS=Homo sapiens GN=KIAA1671 PE=1 SV=2 - [K1671_HUMAN]	3.55	1.33	2	196.6	8.47
Q13492	Phosphatidylinositol-binding clathrin assembly protein OS=Homo sapiens GN=PICALM PE=1 SV=2 - [PICAL_HUMAN]	3.36	1.84	1	70.7	7.90

O00482	Nuclear receptor subfamily 5 group A member 2 OS=Homo sapiens GN=NR5A2 PE=1 SV=2 - [NR5A2_HUMAN]	3.22	6.10	1	61.3	7.88
Q9P2U8	Vesicular glutamate transporter 2 OS=Homo sapiens GN=SLC17A6 PE=2 SV=1 - [VGLU2_HUMAN]	2.82	3.61	1	64.4	7.05
Q92793	CREB-binding protein OS=Homo sapiens GN=CREBBP PE=1 SV=3 - [CBP_HUMAN]	2.65	0.61	1	265.2	8.53
Q02224	Centromere-associated protein E OS=Homo sapiens GN=CENPE PE=1 SV=2 - [CENPE_HUMAN]	2.64	0.41	1	316.2	5.64
Q66K14	TBC1 domain family member 9B OS=Homo sapiens GN=TBC1D9B PE=1 SV=3 - [TBC9B_HUMAN]	2.61	0.64	1	140.4	5.25
Q6N021	Methylcytosine dioxygenase TET2 OS=Homo sapiens GN=TET2 PE=1 SV=3 - [TET2_HUMAN]	2.60	0.80	1	223.7	7.99
Q9NZB2	Constitutive coactivator of PPAR-gamma-like protein 1 OS=Homo sapiens GN=FAM120A PE=1 SV=2 - [F120A_HUMAN]	2.59	1.16	1	121.8	8.88
Q9BYP7	Serine/threonine-protein kinase WNK3 OS=Homo sapiens GN=WNK3 PE=1 SV=3 - [WNK3_HUMAN]	2.59	1.61	1	198.3	6.07
B0I1T2	Unconventional myosin-Ig OS=Homo sapiens GN=MYO1G PE=1 SV=2 - [MYO1G_HUMAN]	2.58	3.63	1	116.4	8.73
Q9P2N5	RNA-binding protein 27 OS=Homo sapiens GN=RBM27 PE=1 SV=2 - [RBM27_HUMAN]	2.53	1.04	1	118.6	9.19
Q4VNC1	Probable cation-transporting ATPase 13A4 OS=Homo sapiens GN=ATP13A4 PE=2 SV=3 - [AT134_HUMAN]	2.42	2.51	1	133.9	6.70
Q8NDW8	Tetratricopeptide repeat protein 21A OS=Homo sapiens GN=TTC21A PE=2 SV=3 - [TT21A_HUMAN]	2.37	0.83	1	150.8	7.24
Q00325	Phosphate carrier protein, mitochondrial OS=Homo sapiens GN=SLC25A3 PE=1 SV=2 - [MPCP_HUMAN]	2.31	3.31	1	40.1	9.38
Q01082	Spectrin beta chain, non-erythrocytic 1 OS=Homo sapiens GN=SPTBN1 PE=1 SV=2 - [SPTB2_HUMAN]	2.31	0.42	1	274.4	5.57
O15320	cTAGE family member 5 OS=Homo sapiens GN=CTAGE5 PE=1 SV=4 - [CTGE5_HUMAN]	2.29	1.74	1	90.9	5.22
Q9ULL5	Proline-rich protein 12 OS=Homo sapiens GN=PRR12 PE=1 SV=2 - [PRR12_HUMAN]	2.20	0.91	1	129.9	8.00
Q92499	ATP-dependent RNA helicase DDX1 OS=Homo sapiens GN=DDX1 PE=1 SV=2 - [DDX1_HUMAN]	2.19	1.35	1	82.4	7.23
Q13085	Acetyl-CoA carboxylase 1 OS=Homo sapiens GN=ACACA PE=1 SV=2 - [ACACA_HUMAN]	2.18	0.47	1	265.4	6.37
O60779	Thiamine transporter 1 OS=Homo sapiens GN=SLC19A2 PE=1 SV=2 - [S19A2_HUMAN]	2.16	2.21	1	55.4	6.74
Q9Y4C1	Lysine-specific demethylase 3A OS=Homo sapiens GN=KDM3A PE=1 SV=4 - [KDM3A_HUMAN]	2.16	0.98	1	147.2	8.07
Q96AY4	Tetratricopeptide repeat protein 28 OS=Homo sapiens GN=TTC28 PE=1 SV=4 - [TTC28_HUMAN]	2.12	0.52	1	270.7	6.89
Q96EV2	RNA-binding protein 33 OS=Homo sapiens GN=RBM33 PE=1 SV=3 - [RBM33_HUMAN]	1.96	1.11	1	129.9	6.93
Q14678	KN motif and ankyrin repeat domain-containing protein 1 OS=Homo sapiens GN=KANK1 PE=1 SV=3 - [KANK1_HUMAN]	1.96	0.67	1	147.2	5.30
Q7Z333	Probable helicase senataxin OS=Homo sapiens GN=SETX PE=1 SV=4 - [SETX_HUMAN]	1.90	0.49	1	302.7	7.17
Q14008	Cytoskeleton-associated protein 5 OS=Homo sapiens GN=CKAP5 PE=1 SV=3 - [CKAP5_HUMAN]	1.84	0.84	2	225.4	7.80
P51587	Breast cancer type 2 susceptibility protein OS=Homo sapiens GN=BRCA2 PE=1 SV=2 - [BRCA2_HUMAN]	1.84	0.35	1	384.0	6.73

P68543	UBX domain-containing protein 2A OS=Homo sapiens GN=UBXN2A PE=2 SV=1 - [UBX2A_HUMAN]	1.81	3.09	1	29.3	6.25
Q9BXT5	Testis-expressed sequence 15 protein OS=Homo sapiens GN=TEX15 PE=2 SV=2 - [TEX15_HUMAN]	1.79	0.25	1	315.1	6.19
P51114	Fragile X mental retardation syndrome-related protein 1 OS=Homo sapiens GN=FXR1 PE=1 SV=3 - [FXR1_HUMAN]	1.79	2.58	2	69.7	6.15
Q15233	Non-POU domain-containing octamer-binding protein OS=Homo sapiens GN=NONO PE=1 SV=4 - [NONO_HUMAN]	1.73	2.97	1	54.2	8.95
Q9BWV2	Spermatogenesis-associated protein 9 OS=Homo sapiens GN=SPATA9 PE=2 SV=2 - [SPAT9_HUMAN]	1.72	2.76	1	28.7	9.48
O15016	Tripartite motif-containing protein 66 OS=Homo sapiens GN=TRIM66 PE=2 SV=4 - [TRI66_HUMAN]	1.70	0.49	1	134.6	6.98
Q8NI27	THO complex subunit 2 OS=Homo sapiens GN=THOC2 PE=1 SV=2 - [THOC2_HUMAN]	1.70	0.56	1	182.7	8.44
P09001	39S ribosomal protein L3, mitochondrial OS=Homo sapiens GN=MRPL3 PE=1 SV=1 - [RM03_HUMAN]	1.67	2.59	1	38.6	9.48
O75899	Gamma-aminobutyric acid type B receptor subunit 2 OS=Homo sapiens GN=GABBR2 PE=1 SV=1 - [GABR2_HUMAN]	1.67	0.85	1	105.8	8.66
Q9Y6N7	Roundabout homolog 1 OS=Homo sapiens GN=ROBO1 PE=1 SV=1 - [ROBO1_HUMAN]	1.65	0.79	1	180.8	6.04
O60423	Phospholipid-transporting ATPase IK OS=Homo sapiens GN=ATP8B3 PE=2 SV=4 - [AT8B3_HUMAN]	0.00	0.54	1	146.7	7.90
P36406	E3 ubiquitin-protein ligase TRIM23 OS=Homo sapiens GN=TRIM23 PE=1 SV=1 - [TRI23_HUMAN]	0.00	1.22	1	64.0	6.38
Q13459	Unconventional myosin-IXb OS=Homo sapiens GN=MYO9B PE=1 SV=3 - [MYO9B_HUMAN]	0.00	0.42	1	243.2	8.75
Q6ZRS4	Coiled-coil domain-containing protein 129 OS=Homo sapiens GN=CCDC129 PE=2 SV=2 - [CC129_HUMAN]	0.00	0.96	1	115.3	5.31
Q7RTP6	Protein-methionine sulfoxide oxidase MICAL3 OS=Homo sapiens GN=MICAL3 PE=1 SV=2 - [MICA3_HUMAN]	0.00	0.40	1	224.2	5.55
Q8WW22	DnaJ homolog subfamily A member 4 OS=Homo sapiens GN=DNAJA4 PE=1 SV=1 - [DNJA4_HUMAN]	0.00	5.29	1	44.8	7.59
Q9BYW2	Histone-lysine N-methyltransferase SETD2 OS=Homo sapiens GN=SETD2 PE=1 SV=3 - [SETD2_HUMAN]	0.00	0.59	1	287.4	6.14
Q9UBC5	Unconventional myosin-Ia OS=Homo sapiens GN=MYO1A PE=1 SV=1 - [MYO1A_HUMAN]	0.00	0.58	1	118.3	9.31

Supplementary Table 2. The list and sequence of peptides with different histone PTMs used in FMRP-1-200 peptide array.

o	o	o	o	o	o	o	o	o	o	o	o	o	o	o	o	o	o	o	o	o	o	o	o	o	o	o	o	o	o	o	o	o	o	o	o	o	o	o	o	o	o	o	o	o	o	o	o	o	o	o
o	**A 1**	**A 2**	**A 3**	**A 4**	**A 5**	**A 6**	**A 7**	**A 8**	**A 9**	**A 10**	**A 11**	**A 12**	**A 13**	**A 14**	**A 15**	**A 16**	**A 17**	**A 18**	**A 19**	**A 20**	**A 21**	**A 22**	**A 23**	**A 24**	o	A 1	A 2	A 3	A 4	A 5	A 6	A 7	A 8	A 9	A10	A11	A12	A13	A14	A15	A16	A17	A18	A19	A20	A21	A22	A23	A24	o
o	**B 1**	**B 2**	**B 3**	**B 4**	**B 5**	**B 6**	**B 7**	**B 8**	**B 9**	**B10**	**B11**	**B12**	**B13**	**B14**	**B15**	**B16**	**B17**	**B18**	**B19**	**B20**	**B21**	**B22**	**B23**	**B24**	o	B 1	B 2	B 3	B 4	B 5	B 6	B 7	B 8	B 9	B10	B11	B12	B13	B14	B15	B16	B17	B18	B19	B20	B21	B22	B23	B24	o
o	**C 1**	**C 2**	**C 3**	**C 4**	**C 5**	**C 6**	**C 7**	**C 8**	**C 9**	**C10**	**C11**	**C12**	**C13**	**C14**	**C15**	**C16**	**C17**	**C18**	**C19**	**C20**	**C21**	**C22**	**C23**	**C24**	o	C 1	C 2	C 3	C 4	C 5	C 6	C 7	C 8	C 9	C10	C11	C12	C13	C14	C15	C16	C17	C18	C19	C20	C21	C22	C23	C24	o
o	**D 1**	**D 2**	**D 3**	**D 4**	**D 5**	**D 6**	**D 7**	**D 8**	**D 9**	**D10**	**D11**	**D12**	**D13**	**D14**	**D15**	**D16**	**D17**	**D18**	**D19**	**D20**	**D21**	**D22**	**D23**	**D24**	o	D 1	D 2	D 3	D 4	D 5	D 6	D 7	D 8	D 9	D10	D11	D12	D13	D14	D15	D16	D17	D18	D19	D20	D21	D22	D23	D24	o
o	**E 1**	**E 2**	**E 3**	**E 4**	**E 5**	**E 6**	**E 7**	**E 8**	**E 9**	**E10**	**E11**	**E12**	**E13**	**E14**	**E15**	**E16**	**E17**	**E18**	**E19**	**E20**	**E21**	**E22**	**E23**	**E24**	o	E 1	E 2	E 3	E 4	E 5	E 6	E 7	E 8	E 9	E10	E11	E12	E13	E14	E15	E16	E17	E18	E19	E20	E21	E22	E23	E24	o
o	**F 1**	**F 2**	**F 3**	**F 4**	**F 5**	**F 6**	**F 7**	**F 8**	**F 9**	**F 10**	**F 11**	**F 12**	**F 13**	**F 14**	**F 15**	**F 16**	**F 17**	**F 18**	**F 19**	**F 20**	**F 21**	**F 22**	**F 23**	**F 24**	o	F 1	F 2	F 3	F 4	F 5	F 6	F 7	F 8	F 9	F10	F11	F12	F13	F14	F15	F16	F17	F18	F19	F20	F21	F22	F23	F24	o
o	**G 1**	**G 2**	**G 3**	**G 4**	**G 5**	**G 6**	**G 7**	**G 8**	**G 9**	**G10**	**G11**	**G12**	**G13**	**G14**	**G15**	**G16**	**G17**	**G18**	**G19**	**G20**	**G21**	**G22**	**G23**	**G24**	o	G 1	G 2	G 3	G 4	G 5	G 6	G 7	G 8	G 9	G10	G11	G12	G13	G14	G15	G16	G17	G18	G19	G20	G21	G22	G23	G24	o
o	**H 1**	**H 2**	**H 3**	**H 4**	**H 5**	**H 6**	**H 7**	**H 8**	**H 9**	**H10**	**H11**	**H12**	**H13**	**H14**	**H15**	**H16**	**H17**	**H18**	**H19**	**H20**	**H21**	**H22**	**H23**	**H24**	o	H 1	H 2	H 3	H 4	H 5	H 6	H 7	H 8	H 9	H10	H11	H12	H13	H14	H15	H16	H17	H18	H19	H20	H21	H22	H23	H24	o
o	**I 1**	**I 2**	**I 3**	**I 4**	**I 5**	**I 6**	**I 7**	**I 8**	**I 9**	**I10**	**I11**	**I12**	**I13**	**I14**	**I15**	**I16**	**I17**	**I18**	**I19**	**I20**	**I21**	**I22**	**I23**	**I24**	o	I 1	I 2	I 3	I 4	I 5	I 6	I 7	I 8	I 9	I10	I11	I12	I13	I14	I15	I16	I17	I18	I19	I20	I21	I22	I23	I24	o
o	**J 1**	**J 2**	**J 3**	**J 4**	**J 5**	**J 6**	**J 7**	**J 8**	**J 9**	**J10**	**J11**	**J12**	**J13**	**J14**	**J15**	**J16**	**J17**	**J18**	**J19**	**J20**	**J21**	**J22**	**J23**	**J24**	o	J 1	J 2	J 3	J 4	J 5	J 6	J 7	J 8	J 9	J10	J11	J12	J13	J14	J15	J16	J17	J18	J19	J20	J21	J22	J23	J24	o
o	**K 1**	**K 2**	**K 3**	**K 4**	**K 5**	**K 6**	**K 7**	**K 8**	**K 9**	**K10**	**K11**	**K12**	**K13**	**K14**	**K15**	**K16**	**K17**	**K18**	**K19**	**K20**	**K21**	**K22**	**K23**	**K24**	o	K 1	K 2	K 3	K 4	K 5	K 6	K 7	K 8	K 9	K10	K11	K12	K13	K14	K15	K16	K17	K18	K19	K20	K21	K22	K23	K24	o
o	**L 1**	**L 2**	**L 3**	**L 4**	**L 5**	**L 6**	**L 7**	**L 8**	**L 9**	**L10**	**L11**	**L12**	**L13**	**L14**	**L15**	**L16**	**L17**	**L18**	**L19**	**L20**	**L21**	**L22**	**L23**	**L24**	o	L 1	L 2	L 3	L 4	L 5	L 6	L 7	L 8	L 9	L10	L11	L12	L13	L14	L15	L16	L17	L18	L19	L20	L21	L22	L23	L24	o
o	**M 1**	**M 2**	**M 3**	**M 4**	**M 5**	**M 6**	**M 7**	**M 8**	**M 9**	**M10**	**M11**	**M12**	**M13**	**M14**	**M15**	**M16**	**M17**	**M18**	**M19**	**M20**	**M21**	**M22**	**M23**	**M24**	o	M 1	M 2	M 3	M 4	M 5	M 6	M 7	M 8	M 9	M10	M11	M12	M13	M14	M15	M16	M17	M18	M19	M20	M21	M22	M23	M24	o
o	**N 1**	**N 2**	**N 3**	**N 4**	**N 5**	**N 6**	**N 7**	**N 8**	**N 9**	**N10**	**N11**	**N12**	**N13**	**N14**	**N15**	**N16**	**N17**	**N18**	**N19**	**N20**	**N21**	**N22**	**N23**	**N24**	o	N 1	N 2	N 3	N 4	N 5	N 6	N 7	N 8	N 9	N10	N11	N12	N13	N14	N15	N16	N17	N18	N19	N20	N21	N22	N23	N24	o
o	**O 1**	**O 2**	**O 3**	**O 4**	**O 5**	**O 6**	**O 7**	**O 8**	**O 9**	**O10**	**O11**	**O12**	**O13**	**O14**	**O15**	**O16**	**O17**	**O18**	**O19**	**O20**	**O21**	**O22**	**O23**	**O24**	o	O 1	O 2	O 3	O 4	O 5	O 6	O 7	O 8	O 9	O10	O11	O12	O13	O14	O15	O16	O17	O18	O19	O20	O21	O22	O23	O24	o
o	**P 1**	**P 2**	**P 3**	**P 4**	**P 5**	**P 6**	**P 7**	**P 8**	**P 9**	**P10**	**P11**	**P12**	**P13**	**P14**	**P15**	**P16**	**P17**	**P18**	**P19**	**P20**	**P21**	**P22**	**P23**	**P24**	o	P 1	P 2	P 3	P 4	P 5	P 6	P 7	P 8	P 9	P10	P11	P12	P13	P14	P15	P16	P17	P18	P19	P20	P21	P22	P23	P24	o
o	o	o	o	o	o	o	o	o	o	o	o	o	o	o	o	o	o	o	o	o	o	o	o	o	o	o	o	o	o	o	o	o	o	o	o	o	o	o	o	o	o	o	o	o	o	o	o	o	o	o

CelluSpots™

LOT: XXXXXX-XXX

bold font = link to peptide sequence

normal font = hold mouse over postion and the peptide sequence will show up after a while

Active Motif
MODified™ Histone Peptide Array*
Catalog Nos. 13001 & 13005

Modification number	*Peptide location*	*Peptide sequence*	*to array*	*name*	*Mod1*	*Mod2*	*Mod 3*	*Mod 4*	*N-terminus*
1	A 1	A R T K Q T A R K S T G G K A P R K Q	►	H3 1-19	unmod				free
2	A 2	A Rme2s T K Q T A R K S T G G K A P R K Q	►	H3 1-19	R2me2s				free
3	A 3	A Rme2a T K Q T A R K S T G G K A P R K Q	►	H3 1-19	R2me2a				free
4	A 4	A Cit T K Q T A R K S T G G K A P R K Q	►	H3 1-19	R2Citr				free
5	A 5	A R pT K Q T A R K S T G G K A P R K Q	►	H3 1-19	T3P				free
6	A 6	A R T Kme1 Q T A R K S T G G K A P R K Q	►	H3 1-19	K4me1				free
7	A 7	A R T Kme2 Q T A R K S T G G K A P R K Q	►	H3 1-19	K4me2				free
8	A 8	A R T Kme3 Q T A R K S T G G K A P R K Q	►	H3 1-19	K4me3				free
9	A 9	A R T Kac Q T A R K S T G G K A P R K Q	►	H3 1-19	K4ac				free
10	A10	A R T K Q T A Rme2s K S T G G K A P R K Q	►	H3 1-19	R8me2s				free
11	A11	A R T K Q T A Rme2a K S T G G K A P R K Q	►	H3 1-19	R8me2a				free
12	A12	A R T K Q T A Cit K S T G G K A P R K Q	►	H3 1-19	R8Citr				free
13	A13	A R T K Q T A R Kme1 S T G G K A P R K Q	►	H3 1-19	K9me1				free
14	A14	A R T K Q T A R Kme2 S T G G K A P R K Q	►	H3 1-19	K9me2				free
15	A15	A R T K Q T A R Kme3 S T G G K A P R K Q	►	H3 1-19	K9me3				free
16	A16	A R T K Q T A R Kac S T G G K A P R K Q	►	H3 1-19	K9ac				free
17	A17	A R T K Q T A R K pS T G G K A P R K Q	►	H3 1-19	S10P				free
18	A18	A R T K Q T A R K S pT G G K A P R K Q	►	H3 1-19	T11P				free
19	A19	A R T K Q T A R K S T G G Kac A P R K Q	►	H3 1-19	K14ac				free
20	A20	A Rme2s pT K Q T A R K S T G G K A P R K Q	►	H3 1-19	R2me2s	T3P			free
21	A21	A Rme2s T Kme1 Q T A R K S T G G K A P R K Q	►	H3 1-19	R2me2s	K4me1			free
22	A22	A Rme2s T Kme2 Q T A R K S T G G K A P R K Q	►	H3 1-19	R2me2s	K4me2			free
23	A23	A Rme2s T Kme3 Q T A R K S T G G K A P R K Q	►	H3 1-19	R2me2s	K4me3			free
24	A24	A Rme2s T Kac Q T A R K S T G G K A P R K Q	►	H3 1-19	R2me2s	K4ac			free
25	B 1	A Rme2a pT K Q T A R K S T G G K A P R K Q	►	H3 1-19	R2me2a	T3P			free
26	B 2	A Rme2a T Kme1 Q T A R K S T G G K A P R K Q	►	H3 1-19	R2me2a	K4me1			free
27	B 3	A Rme2a T Kme2 Q T A R K S T G G K A P R K Q	►	H3 1-19	R2me2a	K4me2			free
28	B 4	A Rme2a T Kme3 Q T A R K S T G G K A P R K Q	►	H3 1-19	R2me2a	K4me3			free
29	B 5	A Rme2a T Kac Q T A R K S T G G K A P R K Q	►	H3 1-19	R2me2a	K4ac			free
30	B 6	A Cit pT K Q T A R K S T G G K A P R K Q	►	H3 1-19	R2Citr	T3P			free
31	B 7	A Cit T Kme1 Q T A R K S T G G K A P R K Q	►	H3 1-19	R2Citr	K4me1			free
32	B 8	A Cit T Kme2 Q T A R K S T G G K A P R K Q	►	H3 1-19	R2Citr	K4me2			free
33	B 9	A Cit T Kme3 Q T A R K S T G G K A P R K Q	►	H3 1-19	R2Citr	K4me3			free
34	B10	A Cit T Kac Q T A R K S T G G K A P R K Q	►	H3 1-19	R2Citr	K4ac			free
35	B11	A R pT Kme1 Q T A R K S T G G K A P R K Q	►	H3 1-19	T3P	K4me1			free
36	B12	A R pT Kme2 Q T A R K S T G G K A P R K Q	►	H3 1-19	T3P	K4me2			free
37	B13	A R pT Kme3 Q T A R K S T G G K A P R K Q	►	H3 1-19	T3P	K4me3			free
38	B14	A R pT Kac Q T A R K S T G G K A P R K Q	►	H3 1-19	T3P	K4ac			free
39	B15	A Rme2s pT Kme1 Q T A R K S T G G K A P R K Q	►	H3 1-19	R2me2s	T3P	K4me1		free
40	B16	A Rme2s pT Kme2 Q T A R K S T G G K A P R K Q	►	H3 1-19	R2me2s	T3P	K4me2		free
41	B17	A Rme2s pT Kme3 Q T A R K S T G G K A P R K Q	►	H3 1-19	R2me2s	T3P	K4me3		free
42	B18	A Rme2s pT Kac Q T A R K S T G G K A P R K Q	►	H3 1-19	R2me2s	T3P	K4ac		free
43	B19	A Rme2a pT Kme1 Q T A R K S T G G K A P R K Q	►	H3 1-19	R2me2a	T3P	K4me1		free
44	B20	A Rme2a pT Kme2 Q T A R K S T G G K A P R K Q	►	H3 1-19	R2me2a	T3P	K4me2		free
45	B21	A Rme2a pT Kme3 Q T A R K S T G G K A P R K Q	►	H3 1-19	R2me2a	T3P	K4me3		free
46	B22	A Rme2a pT Kac Q T A R K S T G G K A P R K Q	►	H3 1-19	R2me2a	T3P	K4ac		free
47	B23	A R T K Q T A Rme2a Kme1 S T G G K A P R K Q	►	H3 1-19	R8me2s	K9me1			free
48	B24	A R T K Q T A Rme2a Kme2 S T G G K A P R K Q	►	H3 1-19	R8me2s	K9me2			free
49	C 1	A R T K Q T A Rme2a Kme3 S T G G K A P R K Q	►	H3 1-19	R8me2s	K9me3			free
50	C 2	A R T K Q T A Rme2a Kac S T G G K A P R K Q	►	H3 1-19	R8me2s	K9ac			free
51	C 3	A R T K Q T A Rme2a K pS T G G K A P R K Q	►	H3 1-19	R8me2s	S10P			free
52	C 4	A R T K Q T A Rme2a K S pT G G K A P R K Q	►	H3 1-19	R8me2s	T11P			free
53	C 5	A R T K Q T A Rme2a Kme1 S T G G K A P R K Q	►	H3 1-19	R8me2a	K9me1			free
54	C 6	A R T K Q T A Rme2a Kme2 S T G G K A P R K Q	►	H3 1-19	R8me2a	K9me2			free
55	C 7	A R T K Q T A Rme2a Kme3 S T G G K A P R K Q	►	H3 1-19	R8me2a	K9me3			free
56	C 8	A R T K Q T A Rme2a Kac S T G G K A P R K Q	►	H3 1-19	R8me2a	K9ac			free
57	C 9	A R T K Q T A Rme2a K pS T G G K A P R K Q	►	H3 1-19	R8me2a	S10P			free
58	C10	A R T K Q T A Rme2a K S pT G G K A P R K Q	►	H3 1-19	R8me2a	T11P			free
59	C11	A R T K Q T A Cit Kme1 S T G G K A P R K Q	►	H3 1-19	R8Citr	K9me1			free
60	C12	A R T K Q T A Cit Kme2 S T G G K A P R K Q	►	H3 1-19	R8Citr	K9me2			free
61	C13	A R T K Q T A Cit Kme3 S T G G K A P R K Q	►	H3 1-19	R8Citr	K9me3			free
62	C14	A R T K Q T A Cit Kac S T G G K A P R K Q	►	H3 1-19	R8Citr	K9ac			free
63	C15	A R T K Q T A Cit K pS T G G K A P R K Q	►	H3 1-19	R8Citr	S10P			free
64	C16	A R T K Q T A Cit K S pT G G K A P R K Q	►	H3 1-19	R8Citr	T11P			free
65	C17	A R T K Q T A R Kme1 pS T G G K A P R K Q	►	H3 1-19	K9me1	S10P			free
66	C18	A R T K Q T A R Kme1 S pT G G K A P R K Q	►	H3 1-19	K9me1	T11P			free
67	C19	A R T K Q T A R Kme1 S T G G Kac A P R K Q	►	H3 1-19	K9me1	K14ac			free
68	C20	A R T K Q T A R Kme2 pS T G G K A P R K Q	►	H3 1-19	K9me2	S10P			free
69	C21	A R T K Q T A R Kme2 S pT G G K A P R K Q	►	H3 1-19	K9me2	T11P			free
70	C22	A R T K Q T A R Kme2 S T G G Kac A P R K Q	►	H3 1-19	K9me2	K14ac			free
71	C23	A R T K Q T A R Kme3 pS T G G K A P R K Q	►	H3 1-19	K9me3	S10P			free
72	C24	A R T K Q T A R Kme3 S pT G G K A P R K Q	►	H3 1-19	K9me3	T11P			free
73	D 1	A R T K Q T A R Kme3 S T G G Kac A P R K Q	►	H3 1-19	K9me3	K14ac			free
74	D 2	A R T K Q T A R Kac pS T G G K A P R K Q	►	H3 1-19	K9ac	S10P			free
75	D 3	A R T K Q T A R Kac S pT G G K A P R K Q	►	H3 1-19	K9ac	T11P			free
76	D 4	A R T K Q T A R Kac S T G G Kac A P R K Q	►	H3 1-19	K9ac	K14ac			free
77	D 5	A R T K Q T A R K pS pT G G K A P R K Q	►	H3 1-19	S10P	T11P			free
78	D 6	A R T K Q T A R K pS T G G Kac A P R K Q	►	H3 1-19	S10P	K14ac			free
79	D 7	A R T K Q T A R K S pT G G Kac A P R K Q	►	H3 1-19	T11P	K14ac			free
80	D 8	A R T K Q T A Rme2s Kme1 pS T G G K A P R K Q	►	H3 1-19	R8me2s	K9me1	S10P		free
81	D 9	A R T K Q T A Rme2s Kme2 pS T G G K A P R K Q	►	H3 1-19	R8me2s	K9me2	S10P		free
82	D10	A R T K Q T A Rme2s Kme3 pS T G G K A P R K Q	►	H3 1-19	R8me2s	K9me3	S10P		free
83	D11	A R T K Q T A Rme2s Kac pS T G G K A P R K Q	►	H3 1-19	R8me2s	K9ac	S10P		free
84	D12	A R T K Q T A Rme2s Kme1 S pT G G K A P R K Q	►	H3 1-19	R8me2s	K9me1	T11P		free
85	D13	A R T K Q T A Rme2s Kme2 S pT G G K A P R K Q	►	H3 1-19	R8me2s	K9me2	T11P		free
86	D14	A R T K Q T A Rme2s Kme3 S pT G G K A P R K Q	►	H3 1-19	R8me2s	K9me3	T11P		free
87	D15	A R T K Q T A Rme2s Kac S pT G G K A P R K Q	►	H3 1-19	R8me2s	K9ac	T11P		free
88	D16	A R T K Q T A Rme2a Kme1 pS T G G K A P R K Q	►	H3 1-19	R8me2a	K9me1	S10P		free
89	D17	A R T K Q T A Rme2a Kme2 pS T G G K A P R K Q	►	H3 1-19	R8me2a	K9me2	S10P		free
90	D18	A R T K Q T A Rme2a Kme3 pS T G G K A P R K Q	►	H3 1-19	R8me2a	K9me3	S10P		free
91	D19	A R T K Q T A Rme2a Kac pS T G G K A P R K Q	►	H3 1-19	R8me2a	K9ac	S10P		free
92	D20	A R T K Q T A Rme2a Kme1 S pT G G K A P R K Q	►	H3 1-19	R8me2a	K9me1	T11P		free
93	D21	A R T K Q T A Rme2a Kme2 S pT G G K A P R K Q	►	H3 1-19	R8me2a	K9me2	T11P		free
94	D22	A R T K Q T A Rme2a Kme3 S pT G G K A P R K Q	►	H3 1-19	R8me2a	K9me3	T11P		free
95	D23	A R T K Q T A Rme2a Kac S pT G G K A P R K Q	►	H3 1-19	R8me2a	K9ac	T11P		free

Active Motif
MODified™ Histone Peptide Array*
Catalog Nos. 13001 & 13005

Modification number	*Peptide location*	*Peptide sequence*	*to array*	*name*	*Mod1*	*Mod2*	*Mod 3*	*Mod 4*	*N-terminus*
96	D24	A R T K Q T A Rme2a Kme1 pS pT G G K A P R K Q	►	H3 1-19	R8me2a	K9me1	S10P	T11P	free
97	E 1	A R T K Q T A Rme2a Kme2 pS pT G G K A P R K Q	►	H3 1-19	R8me2a	K9me2	S10P	T11P	free
98	E 2	A R T K Q T A Rme2a Kme3 pS pT G G K A P R K Q	►	H3 1-19	R8me2a	K9me3	S10P	T11P	free
99	E 3	A R T K Q T A Rme2a Kac pS pT G G K A P R K Q	►	H3 1-19	R8me2a	K9ac	S10P	T11P	free
100	E 4	A Rme2s T Kme1 Q T A Rme2s K S T G G K A P R K Q	►	H3 1-19	R2me2s	K4me1	R8me2s		free
101	E 5	A Rme2s T Kme2 Q T A Rme2s K S T G G K A P R K Q	►	H3 1-19	R2me2s	K4me2	R8me2s		free
102	E 6	A Rme2s T Kme3 Q T A Rme2s K S T G G K A P R K Q	►	H3 1-19	R2me2s	K4me3	R8me2s		free
103	E 7	A Rme2s T Kac Q T A Rme2s K S T G G K A P R K Q	►	H3 1-19	R2me2s	K4ac	R8me2s		free
104	E 8	A Rme2a T Kme1 Q T A Rme2a K S T G G K A P R K Q	►	H3 1-19	R2me2a	K4me1	R8me2a		free
105	E 9	A Rme2a T Kme2 Q T A Rme2a K S T G G K A P R K Q	►	H3 1-19	R2me2a	K4me2	R8me2a		free
106	E10	A Rme2a T Kme3 Q T A Rme2a K S T G G K A P R K Q	►	H3 1-19	R2me2a	K4me3	R8me2a		free
107	E11	A Rme2a T Kac Q T A Rme2a K S T G G K A P R K Q	►	H3 1-19	R2me2a	K4ac	R8me2a		free
108	E12	A Rme2s T Kme1 Q T A R Kme1 S T G G K A P R K Q	►	H3 1-19	R2me2s	K4me1	K9me1		free
109	E13	A Rme2s T Kme2 Q T A R Kme1 S T G G K A P R K Q	►	H3 1-19	R2me2s	K4me2	K9me1		free
110	E14	A Rme2s T Kme3 Q T A R Kme1 S T G G K A P R K Q	►	H3 1-19	R2me2s	K4me3	K9me1		free
111	E15	A Rme2s T Kac Q T A R Kme1 S T G G K A P R K Q	►	H3 1-19	R2me2s	K4ac	K9me1		free
112	E16	A Rme2a T Kme1 Q T A R Kme2 S T G G K A P R K Q	►	H3 1-19	R2me2a	K4me1	K9me2		free
113	E17	A Rme2a T Kme2 Q T A R Kme2 S T G G K A P R K Q	►	H3 1-19	R2me2a	K4me2	K9me2		free
114	E18	A Rme2a T Kme3 Q T A R Kme2 S T G G K A P R K Q	►	H3 1-19	R2me2a	K4me3	K9me2		free
115	E19	A Rme2a T Kac Q T A R Kme2 S T G G K A P R K Q	►	H3 1-19	R2me2a	K4ac	K9me2		free
116	E20	A Rme2s T Kme1 Q T A R Kme3 S T G G K A P R K Q	►	H3 1-19	R2me2s	K4me1	K9me3		free
117	E21	A Rme2s T Kme2 Q T A R Kme3 S T G G K A P R K Q	►	H3 1-19	R2me2s	K4me2	K9me3		free
118	E22	A Rme2s T Kme3 Q T A R Kme3 S T G G K A P R K Q	►	H3 1-19	R2me2s	K4me3	K9me3		free
119	E23	A Rme2s T Kac Q T A R Kme3 S T G G K A P R K Q	►	H3 1-19	R2me2s	K4ac	K9me3		free
120	E24	A Rme2a T Kme1 Q T A R Kac S T G G K A P R K Q	►	H3 1-19	R2me2a	K4me1	K9ac		free
121	F 1	A Rme2a T Kme2 Q T A R Kac S T G G K A P R K Q	►	H3 1-19	R2me2a	K4me2	K9ac		free
122	F 2	A Rme2a T Kme3 Q T A R Kac S T G G K A P R K Q	►	H3 1-19	R2me2a	K4me3	K9ac		free
123	F 3	A Rme2a T Kac Q T A R Kac S T G G K A P R K Q	►	H3 1-19	R2me2a	K4ac	K9ac		free
124	F 4	A R T Kme1 Q T A Rme2s Kme1 S T G G K A P R K Q	►	H3 1-19	K4me1	R8me2s	K9me1		free
125	F 5	A R T Kme2 Q T A Rme2s Kme1 S T G G K A P R K Q	►	H3 1-19	K4me2	R8me2s	K9me1		free
126	F 6	A R T Kme3 Q T A Rme2s Kme1 S T G G K A P R K Q	►	H3 1-19	K4me3	R8me2s	K9me1		free
127	F 7	A R T Kac Q T A Rme2s Kme1 S T G G K A P R K Q	►	H3 1-19	K4ac	R8me2s	K9me1		free
128	F 8	A R T Kme1 Q T A Rme2a Kme1 S T G G K A P R K Q	►	H3 1-19	K4me1	R8me2a	K9me1		free
129	F 9	A R T Kme2 Q T A Rme2a Kme1 S T G G K A P R K Q	►	H3 1-19	K4me2	R8me2a	K9me1		free
130	F10	A R T Kme3 Q T A Rme2a Kme1 S T G G K A P R K Q	►	H3 1-19	K4me3	R8me2a	K9me1		free
131	F11	A R T Kac Q T A Rme2a Kme1 S T G G K A P R K Q	►	H3 1-19	K4ac	R8me2a	K9me1		free
132	F12	A R T Kme1 Q T A Rme2s Kme2 S T G G K A P R K Q	►	H3 1-19	K4me1	R8me2s	K9me2		free
133	F13	A R T Kme2 Q T A Rme2s Kme2 S T G G K A P R K Q	►	H3 1-19	K4me2	R8me2s	K9me2		free
134	F14	A R T Kme3 Q T A Rme2s Kme2 S T G G K A P R K Q	►	H3 1-19	K4me3	R8me2s	K9me2		free
135	F15	A R T Kac Q T A Rme2s Kme2 S T G G K A P R K Q	►	H3 1-19	K4ac	R8me2s	K9me2		free
136	F16	A R T Kme1 Q T A Rme2a Kme2 S T G G K A P R K Q	►	H3 1-19	K4me1	R8me2a	K9me2		free
137	F17	A R T Kme2 Q T A Rme2a Kme2 S T G G K A P R K Q	►	H3 1-19	K4me2	R8me2a	K9me2		free
138	F18	A R T Kme3 Q T A Rme2a Kme2 S T G G K A P R K Q	►	H3 1-19	K4me3	R8me2a	K9me2		free
139	F19	A R T Kac Q T A Rme2a Kme2 S T G G K A P R K Q	►	H3 1-19	K4ac	R8me2a	K9me2		free
140	F20	A R T Kme1 Q T A Rme2s Kme3 S T G G K A P R K Q	►	H3 1-19	K4me1	R8me2s	K9me3		free
141	F21	A R T Kme2 Q T A Rme2s Kme3 S T G G K A P R K Q	►	H3 1-19	K4me2	R8me2s	K9me3		free
142	F22	A R T Kme3 Q T A Rme2s Kme3 S T G G K A P R K Q	►	H3 1-19	K4me3	R8me2s	K9me3		free
143	F23	A R T Kac Q T A Rme2s Kme3 S T G G K A P R K Q	►	H3 1-19	K4ac	R8me2s	K9me3		free
144	F24	A R T Kme1 Q T A Rme2a Kme3 S T G G K A P R K Q	►	H3 1-19	K4me1	R8me2a	K9me3		free
145	G 1	A R T Kme2 Q T A Rme2a Kme3 S T G G K A P R K Q	►	H3 1-19	K4me2	R8me2a	K9me3		free
146	G 2	A R T Kme3 Q T A Rme2a Kme3 S T G G K A P R K Q	►	H3 1-19	K4me3	R8me2a	K9me3		free
147	G 3	A R T Kac Q T A Rme2a Kme3 S T G G K A P R K Q	►	H3 1-19	K4ac	R8me2a	K9me3		free
148	G 4	A R T Kme1 Q T A Rme2s Kac S T G G K A P R K Q	►	H3 1-19	K4me1	R8me2s	K9ac		free
149	G 5	A R T Kme2 Q T A Rme2s Kac S T G G K A P R K Q	►	H3 1-19	K4me2	R8me2s	K9ac		free
150	G 6	A R T Kme3 Q T A Rme2s Kac S T G G K A P R K Q	►	H3 1-19	K4me3	R8me2s	K9ac		free
151	G 7	A R T Kac Q T A Rme2s Kac S T G G K A P R K Q	►	H3 1-19	K4ac	R8me2s	K9ac		free
152	G 8	A R T Kme1 Q T A Rme2a Kac S T G G K A P R K Q	►	H3 1-19	K4me1	R8me2a	K9ac		free
153	G 9	A R T Kme2 Q T A Rme2a Kac S T G G K A P R K Q	►	H3 1-19	K4me2	R8me2a	K9ac		free
154	G10	A R T Kme3 Q T A Rme2a Kac S T G G K A P R K Q	►	H3 1-19	K4me3	R8me2a	K9ac		free
155	G11	A R T Kac Q T A Rme2a Kac S T G G K A P R K Q	►	H3 1-19	K4ac	R8me2a	K9ac		free
156	G12	A Rme2s T Kme1 Q T A Rme2s Kme1 S T G G K A P R K Q	►	H3 1-19	R2me2s	K4me1	R8me2s	K9me1	free
157	G13	A Rme2s T Kme2 Q T A Rme2s Kme1 S T G G K A P R K Q	►	H3 1-19	R2me2s	K4me2	R8me2s	K9me1	free
158	G14	A Rme2s T Kme3 Q T A Rme2s Kme1 S T G G K A P R K Q	►	H3 1-19	R2me2s	K4me3	R8me2s	K9me1	free
159	G15	A Rme2s T Kac Q T A Rme2s Kme1 S T G G K A P R K Q	►	H3 1-19	R2me2s	K4ac	R8me2s	K9me1	free
160	G16	A Rme2a T Kme1 Q T A Rme2s Kme1 S T G G K A P R K Q	►	H3 1-19	R2me2a	K4me1	R8me2s	K9me1	free
161	G17	A Rme2a T Kme2 Q T A Rme2s Kme1 S T G G K A P R K Q	►	H3 1-19	R2me2a	K4me2	R8me2s	K9me1	free
162	G18	A Rme2a T Kme3 Q T A Rme2s Kme1 S T G G K A P R K Q	►	H3 1-19	R2me2a	K4me3	R8me2s	K9me1	free
163	G19	A Rme2a T Kac Q T A Rme2s Kme1 S T G G K A P R K Q	►	H3 1-19	R2me2a	K4ac	R8me2s	K9me1	free
164	G20	A Rme2s T Kme1 Q T A Rme2s Kme2 S T G G K A P R K Q	►	H3 1-19	R2me2s	K4me1	R8me2s	K9me2	free
165	G21	A Rme2s T Kme2 Q T A Rme2s Kme2 S T G G K A P R K Q	►	H3 1-19	R2me2s	K4me2	R8me2s	K9me2	free
166	G22	A Rme2s T Kme3 Q T A Rme2s Kme2 S T G G K A P R K Q	►	H3 1-19	R2me2s	K4me3	R8me2s	K9me2	free
167	G23	A Rme2s T Kac Q T A Rme2s Kme2 S T G G K A P R K Q	►	H3 1-19	R2me2s	K4ac	R8me2s	K9me2	free
168	G24	A Rme2a T Kme1 Q T A Rme2s Kme2 S T G G K A P R K Q	►	H3 1-19	R2me2a	K4me1	R8me2s	K9me2	free
169	H 1	A Rme2a T Kme2 Q T A Rme2s Kme2 S T G G K A P R K Q	►	H3 1-19	R2me2a	K4me2	R8me2s	K9me2	free
170	H 2	A Rme2a T Kme3 Q T A Rme2s Kme2 S T G G K A P R K Q	►	H3 1-19	R2me2a	K4me3	R8me2s	K9me2	free
171	H 3	A Rme2a T Kac Q T A Rme2s Kme2 S T G G K A P R K Q	►	H3 1-19	R2me2a	K4ac	R8me2s	K9me2	free
172	H 4	A Rme2s T Kme1 Q T A Rme2s Kme3 S T G G K A P R K Q	►	H3 1-19	R2me2s	K4me1	R8me2s	K9me3	free
173	H 5	A Rme2s T Kme2 Q T A Rme2s Kme3 S T G G K A P R K Q	►	H3 1-19	R2me2s	K4me2	R8me2s	K9me3	free
174	H 6	A Rme2s T Kme3 Q T A Rme2s Kme3 S T G G K A P R K Q	►	H3 1-19	R2me2s	K4me3	R8me2s	K9me3	free
175	H 7	A Rme2s T Kac Q T A Rme2s Kme3 S T G G K A P R K Q	►	H3 1-19	R2me2s	K4ac	R8me2s	K9me3	free
176	H 8	A Rme2a T Kme1 Q T A Rme2s Kme3 S T G G K A P R K Q	►	H3 1-19	R2me2a	K4me1	R8me2s	K9me3	free
177	H 9	A Rme2a T Kme2 Q T A Rme2s Kme3 S T G G K A P R K Q	►	H3 1-19	R2me2a	K4me2	R8me2s	K9me3	free
178	H10	A Rme2a T Kme3 Q T A Rme2s Kme3 S T G G K A P R K Q	►	H3 1-19	R2me2a	K4me3	R8me2s	K9me3	free
179	H11	A Rme2a T Kac Q T A Rme2s Kme3 S T G G K A P R K Q	►	H3 1-19	R2me2a	K4ac	R8me2s	K9me3	free
180	H12	A Rme2s T Kme1 Q T A Rme2s Kac S T G G K A P R K Q	►	H3 1-19	R2me2s	K4me1	R8me2s	K9ac	free
181	H13	A Rme2s T Kme2 Q T A Rme2s Kac S T G G K A P R K Q	►	H3 1-19	R2me2s	K4me2	R8me2s	K9ac	free
182	H14	A Rme2s T Kme3 Q T A Rme2s Kac S T G G K A P R K Q	►	H3 1-19	R2me2s	K4me3	R8me2s	K9ac	free
183	H15	A Rme2s T Kac Q T A Rme2s Kac S T G G K A P R K Q	►	H3 1-19	R2me2s	K4ac	R8me2s	K9ac	free
184	H16	A Rme2a T Kme1 Q T A Rme2s Kac S T G G K A P R K Q	►	H3 1-19	R2me2a	K4me1	R8me2s	K9ac	free
185	H17	A Rme2a T Kme2 Q T A Rme2s Kac S T G G K A P R K Q	►	H3 1-19	R2me2a	K4me2	R8me2s	K9ac	free
186	H18	A Rme2a T Kme3 Q T A Rme2s Kac S T G G K A P R K Q	►	H3 1-19	R2me2a	K4me3	R8me2s	K9ac	free
187	H19	A Rme2a T Kac Q T A Rme2s Kac S T G G K A P R K Q	►	H3 1-19	R2me2a	K4ac	R8me2s	K9ac	free
188	H20	A Rme2s T Kme1 Q T A Rme2a Kme1 S T G G K A P R K Q	►	H3 1-19	R2me2s	K4me1	R8me2a	K9me1	free
189	H21	A Rme2s T Kme2 Q T A Rme2a Kme1 S T G G K A P R K Q	►	H3 1-19	R2me2s	K4me2	R8me2a	K9me1	free
190	H22	A Rme2s T Kme3 Q T A Rme2a Kme1 S T G G K A P R K Q	►	H3 1-19	R2me2s	K4me3	R8me2a	K9me1	free

Modification number	*Peptide location*	*Peptide sequence*	*to array*	*name*	*Mod1*	*Mod2*	*Mod 3*	*Mod 4*	*N-terminus*
191	H23	A Rme2s T Kac Q T A Rme2a Kme1 S T G G K A P R K Q	►	H3 1-19	R2me2s	K4ac	R8me2a	K9me1	free
192	H24	A Rme2a T Kme1 Q T A Rme2a Kme1 S T G G K A P R K Q	►	H3 1-19	R2me2a	K4me1	R8me2a	K9me1	free
193	I 1	A Rme2a T Kme2 Q T A Rme2a Kme1 S T G G K A P R K Q	►	H3 1-19	R2me2a	K4me2	R8me2a	K9me1	free
194	I 2	A Rme2a T Kme3 Q T A Rme2a Kme1 S T G G K A P R K Q	►	H3 1-19	R2me2a	K4me3	R8me2a	K9me1	free
195	I 3	A Rme2a T Kac Q T A Rme2a Kme1 S T G G K A P R K Q	►	H3 1-19	R2me2a	K4ac	R8me2a	K9me1	free
196	I 4	A Rme2s T Kme1 Q T A Rme2a Kme2 S T G G K A P R K Q	►	H3 1-19	R2me2s	K4me1	R8me2a	K9me2	free
197	I 5	A Rme2s T Kme2 Q T A Rme2a Kme2 S T G G K A P R K Q	►	H3 1-19	R2me2s	K4me2	R8me2a	K9me2	free
198	I 6	A Rme2s T Kme3 Q T A Rme2a Kme2 S T G G K A P R K Q	►	H3 1-19	R2me2s	K4me3	R8me2a	K9me2	free
199	I 7	A Rme2s T Kac Q T A Rme2a Kme2 S T G G K A P R K Q	►	H3 1-19	R2me2s	K4ac	R8me2a	K9me2	free
200	I 8	A Rme2a T Kme1 Q T A Rme2a Kme2 S T G G K A P R K Q	►	H3 1-19	R2me2a	K4me1	R8me2a	K9me2	free
201	I 9	A Rme2a T Kme2 Q T A Rme2a Kme2 S T G G K A P R K Q	►	H3 1-19	R2me2a	K4me2	R8me2a	K9me2	free
202	I10	A Rme2a T Kme3 Q T A Rme2a Kme2 S T G G K A P R K Q	►	H3 1-19	R2me2a	K4me3	R8me2a	K9me2	free
203	I11	A Rme2a T Kac Q T A Rme2a Kme2 S T G G K A P R K Q	►	H3 1-19	R2me2a	K4ac	R8me2a	K9me2	free
204	I12	A Rme2s T Kme1 Q T A Rme2a Kme3 S T G G K A P R K Q	►	H3 1-19	R2me2s	K4me1	R8me2a	K9me3	free
205	I13	A Rme2s T Kme2 Q T A Rme2a Kme3 S T G G K A P R K Q	►	H3 1-19	R2me2s	K4me2	R8me2a	K9me3	free
206	I14	A Rme2s T Kme3 Q T A Rme2a Kme3 S T G G K A P R K Q	►	H3 1-19	R2me2s	K4me3	R8me2a	K9me3	free
207	I15	A Rme2s T Kac Q T A Rme2a Kme3 S T G G K A P R K Q	►	H3 1-19	R2me2s	K4ac	R8me2a	K9me3	free
208	I16	A Rme2a T Kme1 Q T A Rme2a Kme3 S T G G K A P R K Q	►	H3 1-19	R2me2a	K4me1	R8me2a	K9me3	free
209	I17	A Rme2a T Kme2 Q T A Rme2a Kme3 S T G G K A P R K Q	►	H3 1-19	R2me2a	K4me2	R8me2a	K9me3	free
210	I18	A Rme2a T Kme3 Q T A Rme2a Kme3 S T G G K A P R K Q	►	H3 1-19	R2me2a	K4me3	R8me2a	K9me3	free
211	I19	A Rme2a T Kac Q T A Rme2a Kme3 S T G G K A P R K Q	►	H3 1-19	R2me2a	K4ac	R8me2a	K9me3	free
212	I20	A Rme2s T Kme1 Q T A Rme2a Kac S T G G K A P R K Q	►	H3 1-19	R2me2s	K4me1	R8me2a	K9ac	free
213	I21	A Rme2s T Kme2 Q T A Rme2a Kac S T G G K A P R K Q	►	H3 1-19	R2me2s	K4me2	R8me2a	K9ac	free
214	I22	A Rme2s T Kme3 Q T A Rme2a Kac S T G G K A P R K Q	►	H3 1-19	R2me2s	K4me3	R8me2a	K9ac	free
215	I23	A Rme2s T Kac Q T A Rme2a Kac S T G G K A P R K Q	►	H3 1-19	R2me2s	K4ac	R8me2a	K9ac	free
216	I24	A Rme2a T Kme1 Q T A Rme2a Kac S T G G K A P R K Q	►	H3 1-19	R2me2a	K4me1	R8me2a	K9ac	free
217	J 1	A Rme2a T Kme2 Q T A Rme2a Kac S T G G K A P R K Q	►	H3 1-19	R2me2a	K4me2	R8me2a	K9ac	free
218	J 2	A Rme2a T Kme3 Q T A Rme2a Kac S T G G K A P R K Q	►	H3 1-19	R2me2a	K4me3	R8me2a	K9ac	free
219	J 3	A Rme2a T Kac Q T A Rme2a Kac S T G G K A P R K Q	►	H3 1-19	R2me2a	K4ac	R8me2a	K9ac	free
220	J 4	A R K S T G G K A P R K Q L A T K A A R	►	H3 7-26	unmod				acetylated
221	J 5	A R K S T G G Kac A P R K Q L A T K A A R	►	H3 7-26	K14ac				acetylated
222	J 6	A R K pS T G G Kac A P R K Q L A T K A A R	►	H3 7-26	K14ac	S10P			acetylated
223	J 7	A R K S pT G G Kac A P R K Q L A T K A A R	►	H3 7-26	K14ac	T11P			acetylated
224	J 8	A R K S T G G K A P Rme2s K Q L A T K A A R	►	H3 7-26	R17me2s				acetylated
225	J 9	A R K S T G G K A P Rme2a K Q L A T K A A R	►	H3 7-26	R17me2a				acetylated
226	J10	A R K S T G G K A P Cit K Q L A T K A A R	►	H3 7-26	R17Citr				acetylated
227	J11	A R K S T G G K A P R Kac Q L A T K A A R	►	H3 7-26	K18ac				acetylated
228	J12	A R K S T G G Kac A P Rme2s K Q L A T K A A R	►	H3 7-26	K14ac	R17me2s			acetylated
229	J13	A R K S T G G Kac A P Rme2a K Q L A T K A A R	►	H3 7-26	K14ac	R17me2a			acetylated
230	J14	A R K S T G G Kac A P R Kac Q L A T K A A R	►	H3 7-26	K14ac	K18ac			acetylated
231	J15	A R K S T G G K A P Rme2s Kac Q L A T K A A R	►	H3 7-26	R17me2s	K18ac			acetylated
232	J16	A R K S T G G K A P Rme2a Kac Q L A T K A A R	►	H3 7-26	R17me2a	K18ac			acetylated
233	J17	A R K S T G G K A P Cit Kac Q L A T K A A R	►	H3 7-26	R17Citr	K18ac			acetylated
234	J18	A R K S T G G Kac A P Rme2s Kac Q L A T K A A R	►	H3 7-26	K14ac	R17me2s	K18ac		acetylated
235	J19	A R K S T G G Kac A P Rme2a Kac Q L A T K A A R	►	H3 7-26	K14ac	R17me2a	K18ac		acetylated
236	J20	P R K Q L A T K A A R K S A P A T G G	►	H3 16-35	unmod				acetylated
237	J21	P R K Q L A T K A A Rme2s K S A P A T G G	►	H3 16-35	R26me2s				acetylated
238	J22	P R K Q L A T K A A Rme2a K S A P A T G G	►	H3 16-35	R26me2a				acetylated
239	J23	P R K Q L A T K A A Cit K S A P A T G G	►	H3 16-35	R26Citr				acetylated
240	J24	P R K Q L A T K A A R Kme1 S A P A T G G	►	H3 16-35	K27me1				acetylated
241	K 1	P R K Q L A T K A A R Kme2 S A P A T G G	►	H3 16-35	K27me2				acetylated
242	K 2	P R K Q L A T K A A R Kme3 S A P A T G G	►	H3 16-35	K27me3				acetylated
243	K 3	P R K Q L A T K A A R Kac S A P A T G G	►	H3 16-35	K27ac				acetylated
244	K 4	P R K Q L A T K A A R K pS A P A T G G	►	H3 16-35	S28P				acetylated
245	K 5	P R K Q L A T K A A Rme2s Kme1 S A P A T G G	►	H3 16-35	R26me2s	K27me1			acetylated
246	K 6	P R K Q L A T K A A Rme2s Kme2 S A P A T G G	►	H3 16-35	R26me2s	K27me2			acetylated
247	K 7	P R K Q L A T K A A Rme2s Kme3 S A P A T G G	►	H3 16-35	R26me2s	K27me3			acetylated
248	K 8	P R K Q L A T K A A Rme2s Kac S A P A T G G	►	H3 16-35	R26me2s	K27ac			acetylated
249	K 9	P R K Q L A T K A A Rme2s K pS A P A T G G	►	H3 16-35	R26me2s	S28P			acetylated
250	K10	P R K Q L A T K A A Rme2a Kme1 S A P A T G G	►	H3 16-35	R26me2a	K27me1			acetylated
251	K11	P R K Q L A T K A A Rme2a Kme2 S A P A T G G	►	H3 16-35	R26me2a	K27me2			acetylated
252	K12	P R K Q L A T K A A Rme2a Kme3 S A P A T G G	►	H3 16-35	R26me2a	K27me3			acetylated
253	K13	P R K Q L A T K A A Rme2a Kac S A P A T G G	►	H3 16-35	R26me2a	K27ac			acetylated
254	K14	P R K Q L A T K A A Rme2a K pS A P A T G G	►	H3 16-35	R26me2a	S28P			acetylated
255	K15	P R K Q L A T K A A Cit Kme1 S A P A T G G	►	H3 16-35	R26Citr	K27me1			acetylated
256	K16	P R K Q L A T K A A Cit Kme2 S A P A T G G	►	H3 16-35	R26Citr	K27me2			acetylated
257	K17	P R K Q L A T K A A Cit Kme3 S A P A T G G	►	H3 16-35	R26Citr	K27me3			acetylated
258	K18	P R K Q L A T K A A Cit K pS A P A T G G	►	H3 16-35	R26Citr	S28P			acetylated
259	K19	P R K Q L A T K A A R Kme1 pS A P A T G G	►	H3 16-35	K27me1	S28P			acetylated
260	K20	P R K Q L A T K A A R Kme2 pS A P A T G G	►	H3 16-35	K27me2	S28P			acetylated
261	K21	P R K Q L A T K A A R Kme3 pS A P A T G G	►	H3 16-35	K27me3	S28P			acetylated
262	K22	P R K Q L A T K A A R Kac pS A P A T G G	►	H3 16-35	K27ac	S28P			acetylated
263	K23	P R K Q L A T K A A Rme2s Kme1 pS A P A T G G	►	H3 16-35	R26me2s	K27me1	S28P		acetylated
264	K24	P R K Q L A T K A A Rme2s Kme2 pS A P A T G G	►	H3 16-35	R26me2s	K27me2	S28P		acetylated
265	L 1	P R K Q L A T K A A Rme2s Kme3 pS A P A T G G	►	H3 16-35	R26me2s	K27me3	S28P		acetylated
266	L 2	P R K Q L A T K A A Rme2s Kac pS A P A T G G	►	H3 16-35	R26me2s	K27ac	S28P		acetylated
267	L 3	P R K Q L A T K A A Rme2a Kme1 pS A P A T G G	►	H3 16-35	R26me2a	K27me1	S28P		acetylated
268	L 4	P R K Q L A T K A A Rme2a Kme2 pS A P A T G G	►	H3 16-35	R26me2a	K27me2	S28P		acetylated
269	L 5	P R K Q L A T K A A Rme2a Kme3 pS A P A T G G	►	H3 16-35	R26me2a	K27me3	S28P		acetylated
270	L 6	P R K Q L A T K A A Rme2a Kac pS A P A T G G	►	H3 16-35	R26me2a	K27ac	S28P		acetylated
271	L 7	R K S A P A T G G V K K P H R Y R P G	►	H3 26-45	unmod				acetylated
272	L 8	R K S A P A T G G V Kme1 K P H R Y R P G	►	H3 26-45	K36me1				acetylated
273	L 9	R K S A P A T G G V Kme2 K P H R Y R P G	►	H3 26-45	K36me2				acetylated
274	L10	R K S A P A T G G V Kme3 K P H R Y R P G	►	H3 26-45	K36me3				acetylated
275	L11	R K S A P A T G G V Kac K P H R Y R P G	►	H3 26-45	K36ac				acetylated
276	L12	S G R G K G G K G L G K G G A K R H R	►	H4 1-19	unmod				free
277	L13	pS G R G K G G K G L G K G G A K R H R	►	H4 1-19	S1P				free
278	L14	S G Rme2s G K G G K G L G K G G A K R H R	►	H4 1-19	R3me2s				free
279	L15	S G Rme2a G K G G K G L G K G G A K R H R	►	H4 1-19	R3me2a				free
280	L16	S G R G Kac G G K G L G K G G A K R H R	►	H4 1-19	K5ac				free
281	L17	S G R G K G G Kac G L G K G G A K R H R	►	H4 1-19	K8ac				free
282	L18	S G R G K G G K G L G Kac G G A K R H R	►	H4 1-19	K12ac				free
283	L19	S G R G K G G K G L G K G G A Kac R H R	►	H4 1-19	K16ac				free
284	L20	pS G Rme2s G K G G K G L G K G G A K R H R	►	H4 1-19	S1P	R3me2s			free
285	L21	pS G Rme2a G K G G K G L G K G G A K R H R	►	H4 1-19	S1P	R3me2a			free

Active Motif
MODified™ Histone Peptide Array*
Catalog Nos. 13001 & 13005

Modification number	*Peptide location*	*Peptide sequence*	*to array*	*name*	*Mod1*	*Mod2*	*Mod 3*	*Mod 4*	*N-terminus*
286	L22	pS G R G Kac G G K G L G K G G A K R H R	▶	H4 1-19	S1P	K5ac			free
287	L23	S G Rme2s G Kac G G K G L G K G G A K R H R	▶	H4 1-19	R3me2s	K5ac			free
288	L24	S G Rme2s G K G G Kac G L G K G G A K R H R	▶	H4 1-19	R3me2s	K8ac			free
289	M 1	S G Rme2a G Kac G G K G L G K G G A K R H R	▶	H4 1-19	R3me2a	K5ac			free
290	M 2	S G Rme2a G K G G Kac G L G K G G A K R H R	▶	H4 1-19	R3me2a	K8ac			free
291	M 3	S G R G Kac G G Kac G L G K G G A K R H R	▶	H4 1-19	K5ac	K8ac			free
292	M 4	S G R G K G G Kac G L G Kac G G A K R H R	▶	H4 1-19	K8ac	K12ac			free
293	M 5	S G R G K G G Kac G L G K G G A Kac R H R	▶	H4 1-19	K8ac	K16ac			free
294	M 6	S G R G K G G K G L G Kac G G A Kac R H R	▶	H4 1-19	K12ac	K16ac			free
295	M 7	pS G Rme2s G Kac G G K G L G K G G A K R H R	▶	H4 1-19	S1P	R3me2s	K5ac		free
296	M 8	pS G Rme2a G Kac G G K G L G K G G A K R H R	▶	H4 1-19	S1P	R3me2a	K5ac		free
297	M 9	S G Rme2s G Kac G G Kac G L G K G G A K R H R	▶	H4 1-19	R3me2s	K5ac	K8ac		free
298	M10	S G Rme2a G Kac G G Kac G L G K G G A K R H R	▶	H4 1-19	R3me2a	K5ac	K8ac		free
299	M11	S G R G Kac G G Kac G L G Kac G G A K R H R	▶	H4 1-19	K5ac	K8ac	K12ac		free
300	M12	S G R G K G G Kac G L G Kac G G A Kac R H R	▶	H4 1-19	K8ac	K12ac	K16ac		free
301	M13	pS G Rme2s G Kac G G Kac G L G K G G A K R H R	▶	H4 1-19	S1P	R3me2s	K5ac	K8ac	free
302	M14	pS G Rme2a G Kac G G Kac G L G K G G A K R H R	▶	H4 1-19	S1P	R3me2a	K5ac	K8ac	free
303	M15	S G Rme2s G Kac G G Kac G L G Kac G G A K R H R	▶	H4 1-19	R3me2s	K5ac	K8ac	K12ac	free
304	M16	S G Rme2a G Kac G G Kac G L G Kac G G A K R H R	▶	H4 1-19	R3me2a	K5ac	K8ac	K12ac	free
305	M17	S G R G Kac G G Kac G L G Kac G G A Kac R H R	▶	H4 1-19	K5ac	K8ac	K12ac	K16ac	free
306	M18	G K G G A K R H R K V L R D N I Q G I T	▶	H4 11-30	unmod				acetylated
307	M19	G Kac G G A K R H R K V L R D N I Q G I T	▶	H4 11-30	K12ac				acetylated
308	M20	G K G G A Kac R H R K V L R D N I Q G I T	▶	H4 11-30	K16ac				acetylated
309	M21	G K G G A K Rme2s H R K V L R D N I Q G I T	▶	H4 11-30	R17me2s				acetylated
310	M22	G K G G A K Rme2a H R K V L R D N I Q G I T	▶	H4 11-30	R17me2a				acetylated
311	M23	G K G G A K R H Rme2s K V L R D N I Q G I T	▶	H4 11-30	R19me2s				acetylated
312	M24	G K G G A K R H Rme2a K V L R D N I Q G I T	▶	H4 11-30	R19me2a				acetylated
313	N 1	G K G G A K R H R Kme1 V L R D N I Q G I T	▶	H4 11-30	K20me1				acetylated
314	N 2	G K G G A K R H R Kme2 V L R D N I Q G I T	▶	H4 11-30	K20me2				acetylated
315	N 3	G K G G A K R H R Kme3 V L R D N I Q G I T	▶	H4 11-30	K20me3				acetylated
316	N 4	G K G G A K R H R Kac V L R D N I Q G I T	▶	H4 11-30	K20ac				acetylated
317	N 5	G K G G A K R H R K V L Rme2a D N I Q G I T	▶	H4 11-30	R24me2a				acetylated
318	N 6	G K G G A K R H R K V L Rme2s D N I Q G I T	▶	H4 11-30	R24me2s				acetylated
319	N 7	G Kac G G A Kac R H R K V L R D N I Q G I T	▶	H4 11-30	K12ac	K16ac			acetylated
320	N 8	G K G G A Kac Rme2s H R K V L R D N I Q G I T	▶	H4 11-30	K16ac	R17me2s			acetylated
321	N 9	G K G G A Kac Rme2a H R K V L R D N I Q G I T	▶	H4 11-30	K16ac	R17me2a			acetylated
322	N10	G K G G A Kac R H Rme2s K V L R D N I Q G I T	▶	H4 11-30	K16ac	R19me2s			acetylated
323	N11	G K G G A Kac R H Rme2a K V L R D N I Q G I T	▶	H4 11-30	K16ac	R19me2a			acetylated
324	N12	G K G G A Kac R H R Kme1 V L R D N I Q G I T	▶	H4 11-30	K16ac	K20me1			acetylated
325	N13	G K G G A Kac R H R Kme2 V L R D N I Q G I T	▶	H4 11-30	K16ac	K20me2			acetylated
326	N14	G K G G A Kac R H R Kme3 V L R D N I Q G I T	▶	H4 11-30	K16ac	K20me3			acetylated
327	N15	G K G G A Kac R H R Kac V L R D N I Q G I T	▶	H4 11-30	K16ac	K20ac			acetylated
328	N16	G Kac G G A Kac R H R Kme1 V L R D N I Q G I T	▶	H4 11-30	K12ac	K16ac	K20me1		acetylated
329	N17	G Kac G G A Kac R H R Kme2 V L R D N I Q G I T	▶	H4 11-30	K12ac	K16ac	K20me2		acetylated
330	N18	G Kac G G A Kac R H R Kme3 V L R D N I Q G I T	▶	H4 11-30	K12ac	K16ac	K20me3		acetylated
331	N19	G Kac G G A Kac R H R Kac V L R D N I Q G I T	▶	H4 11-30	K12ac	K16ac	K20ac		acetylated
332	N20	G K G G A K R H Rme2a Kme1 V L R D N I Q G I T	▶	H4 11-30	R19me2a	K20me1			acetylated
333	N21	G K G G A K R H Rme2a Kme2 V L R D N I Q G I T	▶	H4 11-30	R19me2a	K20me2			acetylated
334	N22	G K G G A K R H Rme2a Kme3 V L R D N I Q G I T	▶	H4 11-30	R19me2a	K20me3			acetylated
335	N23	G K G G A K R H Rme2a Kac V L R D N I Q G I T	▶	H4 11-30	R19me2a	K20ac			acetylated
336	N24	G K G G A K R H Rme2s Kme1 V L R D N I Q G I T	▶	H4 11-30	R19me2s	K20me1			acetylated
337	O 1	G K G G A K R H Rme2s Kme2 V L R D N I Q G I T	▶	H4 11-30	R19me2s	K20me2			acetylated
338	O 2	G K G G A K R H Rme2s Kme3 V L R D N I Q G I T	▶	H4 11-30	R19me2s	K20me3			acetylated
339	O 3	G K G G A K R H Rme2s Kac V L R D N I Q G I T	▶	H4 11-30	R19me2s	K20ac			acetylated
340	O 4	G K G G A K R H R Kme1 V L Rme2a D N I Q G I T	▶	H4 11-30	R24me2a	K20me1			acetylated
341	O 5	G K G G A K R H R Kme2 V L Rme2a D N I Q G I T	▶	H4 11-30	R24me2a	K20me2			acetylated
342	O 6	G K G G A K R H R Kme3 V L Rme2a D N I Q G I T	▶	H4 11-30	R24me2a	K20me3			acetylated
343	O 7	G K G G A K R H R Kac V L Rme2a D N I Q G I T	▶	H4 11-30	R24me2a	K20ac			acetylated
344	O 8	G K G G A K R H R Kme1 V L Rme2s D N I Q G I T	▶	H4 11-30	R24me2s	K20me1			acetylated
345	O 9	G K G G A K R H R Kme2 V L Rme2s D N I Q G I T	▶	H4 11-30	R24me2s	K20me2			acetylated
346	O10	G K G G A K R H R Kme3 V L Rme2s D N I Q G I T	▶	H4 11-30	R24me2s	K20me3			acetylated
347	O11	G K G G A K R H R Kac V L Rme2s D N I Q G I T	▶	H4 11-30	R24me2s	K20ac			acetylated
348	O12	S G R G K Q G G K A R A K A K S R S S	▶	H2a 1-19	unmod				free
349	O13	pS G R G K Q G G K A R A K A K S R S S	▶	H2a 1-19	S1P				free
350	O14	S G R G Kac Q G G K A R A K A K S R S S	▶	H2a 1-19	K5ac				free
351	O15	S G R G K Q G G Kac A R A K A K S R S S	▶	H2a 1-19	K9ac				free
352	O16	S G R G K Q G G K A R A Kac A K S R S S	▶	H2a 1-19	K13ac				free
353	O17	pS G R G Kac Q G G K A R A K A K S R S S	▶	H2a 1-19	S1P	K5ac			free
354	O18	pS G R G K Q G G Kac A R A K A K S R S S	▶	H2a 1-19	S1P	K9ac			free
355	O19	pS G R G K Q G G K A R A Kac A K S R S S	▶	H2a 1-19	S1P	K13ac			free
356	O20	S G R G Kac Q G G Kac A R A K A K S R S S	▶	H2a 1-19	K5ac	K9ac			free
357	O21	S G R G Kac Q G G K A R A Kac A K S R S S	▶	H2a 1-19	K5ac	K13ac			free
358	O22	S G R G K Q G G Kac A R A Kac A K S R S S	▶	H2a 1-19	K9ac	K13ac			free
359	O23	pS G R G Kac Q G G Kac A R A K A K S R S S	▶	H2a 1-19	S1P	K5ac	K9ac		free
360	O24	pS G R G Kac Q G G K A R A Kac A K S R S S	▶	H2a 1-19	S1P	K5ac	K13ac		free
361	P 1	pS G R G K Q G G Kac A R A Kac A K S R S S	▶	H2a 1-19	S1P	K9ac	K13ac		free
362	P 2	S G R G Kac Q G G Kac A R A Kac A K S R S S	▶	H2a 1-19	K5ac	K9ac	K13ac		free
363	P 3	pS G R G Kac Q G G Kac A R A Kac A K S R S S	▶	H2a 1-19	S1P	K5ac	K9ac	K13ac	free
364	P 4	P D P A K S A P A P K K G S K K A V T	▶	H2B 1-19	unmod				free
365	P 5	P D P A Kac S A P A P K K G S K K A V T	▶	H2B 1-19	K5ac				free
366	P 6	P D P A K S A P A P K Kac G S K K A V T	▶	H2B 1-19	K12ac				free
367	P 7	P D P A K S A P A P K K G pS K K A V T	▶	H2B 1-19	S14P				free
368	P 8	P D P A K S A P A P K K G S Kac K A V T	▶	H2B 1-19	K15ac				free
369	P 9	P D P A Kac S A P A P K Kac G S K K A V T	▶	H2B 1-19	K5ac	K12ac			free
370	P10	P D P A Kac S A P A P K K G pS K K A V T	▶	H2B 1-19	K5ac	S14P			free
371	P11	P D P A Kac S A P A P K K G S Kac K A V T	▶	H2B 1-19	K5ac	K15ac			free
372	P12	P D P A K S A P A P K Kac G pS K K A V T	▶	H2B 1-19	K12ac	S14P			free
373	P13	P D P A K S A P A P K Kac G S Kac K A V T	▶	H2B 1-19	K12ac	K15ac			free
374	P14	P D P A K S A P A P K K G pS Kac K A V T	▶	H2B 1-19	S14P	K15ac			free
375	P15	P D P A Kac S A P A P K Kac G pS K K A V T	▶	H2B 1-19	K5ac	K12ac	S14P		free
376	P16	P D P A Kac S A P A P K Kac G S Kac K A V T	▶	H2B 1-19	K5ac	K12ac	K15ac		free
377	P17	P D P A Kac S A P A P K K G pS Kac K A V T	▶	H2B 1-19	K5ac	S14P	K15ac		free
378	P18	P D P A K S A P A P K Kac G pS Kac K A V T	▶	H2B 1-19	K12ac	S14P	K15ac		free
379	P19	P D P A Kac S A P A P K Kac G pS Kac K A V T	▶	H2B 1-19	K5ac	K12ac	S14P	K15ac	free
380	P20	Bio A A N W S H P Q F E K A A	▶	Biotin, control peptide					biotinylated

Active Motif
MODified™ Histone Peptide Array*
Catalog Nos. 13001 & 13005

Modification number	*Peptide location*	*Peptide sequence*	*to array*	*name*	*Mod1*	*Mod2*	*Mod 3*	*Mod 4*	*N-terminus*
381	P21	E Q K L I S E E D L A	▶	c-myc tag					free
382	P22	HAc	▶	neg. contol					acetylated
383	P23	K Kme1 Kme2 Kme3 Kac R Rme2s R Rme2a R Cit K Kme1 Kac Kme	▶	background 01					acetylated
384	P24	R Rme2s K Kme1 Kac R Rme2a Kme2 K Kme3 R Kme1 Rme2s K Kac	▶	background 02					acetylated

*CelluSpots™ arrays are manufactured under license by INTAVIS Bioanalytical Instruments AG
